# Tritrophic interactions follow phylogenetic escalation and climatic adaptation

**DOI:** 10.1038/s41598-020-59068-2

**Published:** 2020-02-07

**Authors:** Alan Kergunteuil, Laureline Humair, Anne-Laure Maire, María Fernanda Moreno-Aguilar, Adrienne Godschalx, Pilar Catalán, Sergio Rasmann

**Affiliations:** 1 Institute of Biology, University of Neuchâtel, Rue Emile-Argand 11, 2000 Neuchâtel, Switzerland; 2 Present Address: INRAE, UMR Laboratoire d’Agronomie et Environnement, Vandoeuvre-lès, 54518 Nancy, France; 3 Botanical Garden Neuchâtel, Chemin du Pertuis-du-Sault 58, 2000 Neuchâtel, Switzerland; 4 Departamento de Ciencias Agrarias y del Medio Natural, Escuela Politécnica Superior de Huesca, Universidad de Zaragoza, Ctra. Cuarte km 1, 22071 Huesca, Spain; 5 Grupo de Bioquímica, Biofísica y Biología Computacional (BIFI, UNIZAR), Unidad Asociada al CSIC, Zaragoza, Spain; 6 Department of Botany, Institute of Biology, Tomsk State University, Lenin Av. 36, Tomsk, 634050 Russia

**Keywords:** Food webs, Evolutionary ecology, Phylogenetics

## Abstract

One major goal in plant evolutionary ecology is to address how and why tritrophic interactions mediated by phytochemical plant defences vary across species, space, and time. In this study, we tested three classical hypotheses about plant defences: (i) the resource-availability hypothesis, (ii) the altitudinal/elevational gradient hypothesis and (iii) the defence escalation hypothesis. For this purpose, predatory soil nematodes were challenged to hunt for root herbivores based on volatile cues from damaged or intact roots of 18 Alpine *Festuca* grass species adapted to distinct climatic niches spanning 2000 meters of elevation. We found that adaptation into harsh, nutrient-limited alpine environments coincided with the production of specific blends of volatiles, highly attractive for nematodes. We also found that recently-diverged taxa exposed to herbivores released higher amounts of volatiles than ancestrally-diverged species. Therefore, our model provides evidence that belowground indirect plant defences associated with tritrophic interactions have evolved under two classical hypotheses in plant ecology. While phylogenetic drivers of volatile emissions point to the defence-escalation hypothesis, plant local adaptation of indirect defences is in line with the resource availability hypothesis.

## Introduction

The predominant function of plant secondary chemistry is to increase tissue toxicity against herbivores^1^. However, it can also enhance plants’ ability to attract natural enemies of the herbivores^2^. Volatile organic compounds (VOCs), for instance, can serve as attractive cues for foraging predators and parasitoids aboveground^3^, as well as entomopathogenic nematodes (EPNs) belowground^4^. When VOCs, by attracting predators, benefit plants by reducing herbivore performance, they act as indirect defence traits and should be under selection^5,6^. While most plants release VOCs constitutively, most VOCs are, as a cost-saving strategy^7^, only induced upon herbivore feeding. Qualitative and quantitative changes in plant volatile emissions following herbivore attacks are assumed to enhance predator attraction^8^. The magnitude, diversity and intensity of the chemical blend have been shown to vary widely across species and also depend on the local biotic and abiotic conditions^8,9^. Therefore, the unique phytochemical defence phenotype of a plant is the result of several interdependent factors relating to the evolutionary history of plant species and their adaptation to the biotic and abiotic environment^10–12^. A current major goal in plant defence ecology is to disentangle the contributions of ecological and phylogenetic drivers of the natural variation in plant defence expression^13^.

Plant defence theories relating to niche occupation suggest that adaptation and speciation into different habitats result in unique plant chemical phenotypes optimized for the local biotic and abiotic conditions^14^. Within this framework, the abiotic-grounded resource availability hypothesis^15^ states that plant defences vary substantially across resource gradients, in which nutrient-poor habitats should select for higher investment in defence to protect precious tissue build-up^14,16^. The biotic-centred latitudinal/elevational gradient hypothesis suggests that plant defence investment should be greater in warmer and more stable regions as biotic interactions such as herbivory are thought to be stronger in such climates^17–19^. Several tests of this hypothesis have indeed shown that plants invest more in defences in tropical regions^20–22^ as well as at low elevation sites^23,24^ compared to temperate and high elevation regions, respectively. Yet, reviews of such tests have yielded mixed results, both along latitude^25^ and elevation^26^, suggesting that the relationship between climate, herbivore pressure, and plant defence level is not constant and linearly-dependent across scales^27^. These difficulties in predicting plant defence expressions based exclusively on local resources, latitude or elevation call for integrative approaches accounting for highly tractable ecological niches and evolutionary histories of plants. Accordingly, co-evolutionary theory stresses the importance of novel key adaptations in driving phylogenetic diversification through time in response to herbivore pressure^28,29^. Ehrlich and Raven’s^30^ co-evolutionary theory proposes a defence escalation hypothesis, in which the evolution of novel traits that promote speciation, such as novel and more potent defence traits, is incremental (and directional) through the diversification of plant clades. Thus, a phylogenetic escalation for more, and more potent defence traits as lineages diversify should be observed^31,32^.

**Figure 1:**
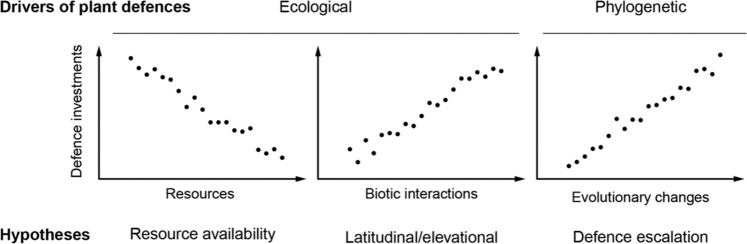
Testing three classical hypotheses for addressing drivers of variation in plant chemical defences against herbivores across scales: from left to right; (1) the resource availability^15^, (2) the latitudinal/elevational gradient ^17,26^, and (3) the defence escalation^30,32^ hypotheses.

We here used the natural elevational gradient of the Alps that provides a range of climate, and natural macro-evolutionary processes to test (i) herbivore effects on VOCs-mediated tritrophic interactions, and (ii) three major hypotheses of classic plant defence theory (Fig. 1). We hypothesized that while classical plant defence theory has been developed for aboveground defence traits directly acting on herbivores (e.g. physical defences such as spines, or chemicals such toxins), its predictions should also hold for indirect defence traits (such as VOCs attracting natural enemies of the herbivores), if these traits confer benefit for the plant^5^, as was recently shown on similar systems^6^. First, we asked if herbivores induce VOCs that facilitate predator recruitment^5^. If the cost of volatile induction remains limited, the ability to induce VOCs after herbivory should be widespread across the genus but the degree of inducibility might vary between species because of their speciation into unique biotic and abiotic environments^33^. Second, we asked if plant speciation within specific local abiotic and biotic conditions shapes VOCs production and nematode recruitment^15,16^. Because litter decomposition and resource availability are reduced under cold temperatures at high elevation, according to the resource availability hypothesis, alpine species should invest more in VOCs production and nematode recruitment. However, if herbivores are the main drivers of VOCs production and subsequent EPN attraction, according to the latitudinal/elevational gradient hypothesis, sites generating higher biotic interactions, such as warmer and more stable low elevation sites, should favour increased VOCs production^17,24,34^. Finally, we asked whether there is evidence for defence escalation as plant lineages diversify^30,32^. If diversification implies a co-evolutionary arms race between plants and herbivores that include predator recruitment, then according to the defence escalation hypothesis, more recently diverged species should produce higher amounts of attractive VOCs involved in tritrophic interactions.

## Results

### Herbivore-induced VOCs facilitate predator recruitment

**Figure 2:**
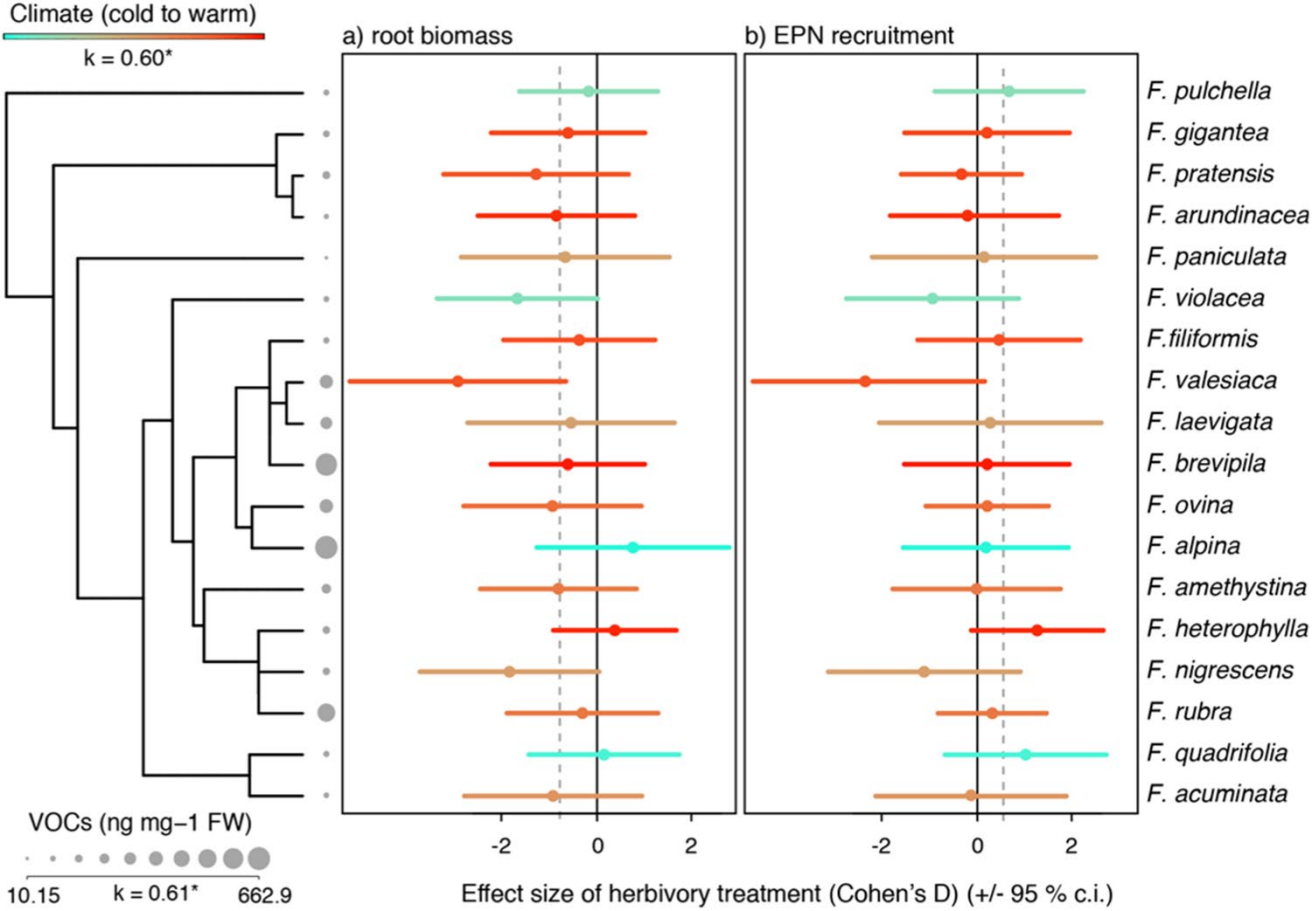
Cladogram showing the Best Maximum Likelihood nuclear ITS tree for the 18 species of *Festuca* studied. The standardized effect sizes (Cohen’s d) for (**a**) root biomass and (**b**) entomopathogenic nematode (EPN) recruitment are shown for each species. The grey dotted line shows the mean effect size across all species (−0.8 for root biomass, and 0.4 for EPN recruitment, respectively). The dots are color-coded according to the optimal climatic niche for each species (Figs. S6 and S7). The climatic niche of each species carries marginal phylogenetic signal (K = 0.60, p = 0.06). Mapped on the tree is the total amount of volatile organic compounds (VOCs) emitted by the roots of the plants after root herbivore induction. Total VOCs carry phylogenetic signal (K = 0.61, p = 0.02).

To address our aims, we studied VOCs production and EPN attraction on plants with and without root herbivore attack across 18 species of *Festuca* that grow in the Alps (Figs. 1, S1). On average, across all species, we found that *Melolontha* herbivory decreased root biomass by about 40% (see supplementary material for description of the statistical methods; Fig. 2a; average 0.8 effect size decrease; and Fig. S2a; MCMCglmm for root biomass; post.mean = −657.9, l–95% CI = −1090.2, u-95% CI = −265.9, sampling = 1000, pMCMC = 0.002), and increased entomopathogenic nematodes (EPNs) recruitment by about 30% when compared to healthy plants (Fig. 2b; 0.48 effect size increase; and Fig. S2b; MCMCglmm for nematodes; post.mean = 96.49 l–5% CI = 31,81, u-95%CI = 160,60, sampling = 868, [31.81, 160.60], pMCMC(868) = 0.002). Predator recruitment is likely mediated by the abundance and identity of herbivore-induced root VOCs released in rhizosphere, which show strong species-level variations (Fig. S3; PERMANOVA; species effect: F_17,148_ = 3.16, p = 0.001). However, we found no effect of the herbivore treatment on volatile profiles (herbivore treatment effect; F_1,148_ = 0.81, p = 0.60; root biomass effect; F_1,148_ = 2.08, p = 0.04, and species by treatment interaction; F_17,148_ = 0.86, p = 0.88), indicating that, although larvae ate abundantly on the roots, root VOCs induction by *M. melolontha* larvae, across the multivariate VOCs matrix, was weak. Herbivore effect on VOCs induction was however strong when assessing the total amount of VOCs produced (Table S4), as well as when assessing individual compounds such as the sesquiterpenoids *E*-β-caryophyllene and isosativene (Table S4, Fig. S4). Finally, we observed a positive association between the total constitutive production of VOCs and their inducibility (Fig. S5; correlation corrected for spurious correlations: r = 0.83, p < 0.001; PGLS, F_1,16_ = 35.82, p < 0.001).

### Plant species adapted to high elevation, cold and humid habitats, recruit more predatory nematodes

**Figure 3:**
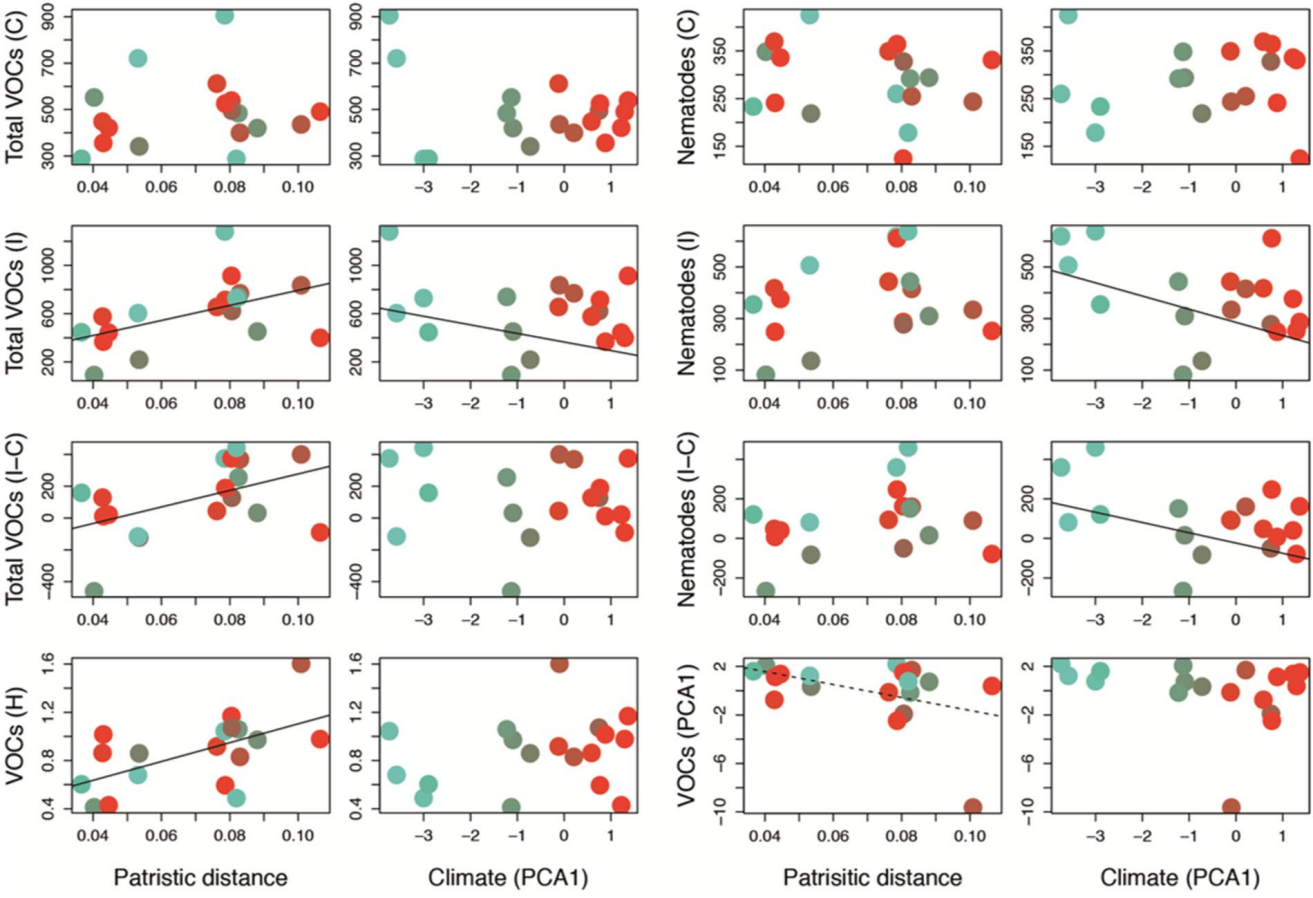
Phylogenetic and climatic effect on root volatile organic compound (VOCs) production and entomopathogenic nematodes (EPNs) attraction across 18 species of *Festuca* growing in the Alps. Each dot represents one species, and dots are color-coded based on their optimal climatic distribution. Green colours represent colder and more humid climates, while red colours represent warmer and drier habitats. Patristic distance represents the total branch length from the last common ancestor, and climate represents the first axis of a principal component analysis (Fig. S6; the axis ranges from negative to positive values, and indicates variation from cold and humid high elevation habitats to warm and dry low elevation habitats). VOCs C are the volatiles emitted by undamaged roots, VOCs I are volatiles emitted by herbivore damaged roots and VOCs I-C is the difference between the two and represents each species root volatile inducibility potential. VOCs H represents the Shannon diversity values. VOCs PCA1 represents the first axis of the PCA ordination of all VOCs emitted at the induced state (Fig. S10). EPNs C indicates the number of EPNs attracted to undamaged roots. EPNs I indicates the number of EPNs attracted to herbivore-damaged roots and EPNs I-C is the difference between the two. The lines show significant correlations (linear model (lm) and phylogenetic corrected model (pgls) for phylogeny and climate, respectively; p < 0.05 for filled lines and p < 0.1 for dotted lines, see Table 1).

The climate of each species’ home site from along the elevational gradient was an important factor determining EPN recruitment. Root herbivore induced-species originating from higher elevation, colder and more humid habitats (Fig. S7) produced almost twice more VOCs and attracted about twice as many nematodes than their lowland congeners from warmer and drier habitats (Fig. 3; see PCA1 axis ranging from negative to positive values, and indicating variation from cold and humid high elevation habitats to warm and dry low elevation habitats, Table 1). Multivariate phylogenetic-corrected regression models of the different climatic variables indicated that the number of precipitation days per growing season (r = 0.7, F_1,16_ = 15.54, p = 0.001), humidity (r = 0.60, F_1,16_ = 9.21, p = 0.01) and number of frost days(r = 0.52, F_1,16_ = 5.85, p = 0.03), which are all intimately associated with elevation, were the best predictors of nematode attraction. Furthermore, we explored the relationship between VOCs production and EPNs attraction with soil variables (Fig. S8, Table S3) and found a significant relationship between herbivore-induced VOCs and soil variables (Fig. S9, distance-based redundancy analysis (dbRDA): F_8,9_ = 1.67, p = 0.01). The soil effect was mainly driven by soil relative humidity (HR), which was also positively associated with the terpenes β-pinene and *E*-β-caryophyllene, while soils richer in organic matter (high CEC, pH and CN values) were negatively associated with the production of the terpenes aromadendrene, β-vatirenene and isosativene. On the other hand, none of the soil variables could explain EPN recruitment measured in the bioassays (overall linear model; r = 0.36, F_8,9_ = 0.18 and p = 0.99).

**Table 1:** Tests for phylogenetic (patristic distance, intervening nodes) and ecological (Climate PCA1) drivers of plant defences. Shown are the results from correlation analyses between patristic distance, number of intervening nodes (lm analyses), and climate (pgls analyses) as response variables on constitutive (C), induced (I), inducible (I-C), Shannon diversity (H), and the structure (PCA) of volatile organic compounds (VOCs) emitted by 18 species of *Festuca* and their respective entomopathogenic nematodes (EPN) recruited. Note that Climate PCA1 ranges from high elevation, cold and humid habitats (negative ordination scores) to low elevation, warm and dry habitats (positive ordination scores). Hence, negative correlations with PCA1 indicate that response variables are more intense in cold and humid Alpine habitats.

Dependent variable	Response variable	r^†^	F_1,16_^†^	p	Response variable	r	F_1,16_	p
Branch length	Tot VOCs C	0.18	0.37	0.55	EPNs C	−0.18	0.56	0.46
	Tot VOCs I	0.46	5.34	0.03**	EPNs I	0.23	0.92	0.35
	Tot VOCs I-C	0.49	4.96	0.04**	EPNs I-C	0.30	1.61	0.22
	PCA VOCs	−0.43	3.61	**0.07°**	EPNs es	−0.13	0.27	0.61
	H VOCs	0.57	7.82	0.01**				
Intervening Nodes	Tot VOCs C	0.31	1.78	0.20	EPNs C	0.06	0.07	0.78
	Tot VOCs I	0.56	7.08	0.02**	EPNs I	0.17	0.52	0.48
	Tot VOCs I-C	0.43	3.87	**0.06°**	EPNs I-C	0.2	0.64	0.43
	PCA VOCs	0.39	2.73	0.12	EPNs es	0.14	0.34	0.56
	H VOCs	0.62	10.55	0.01**				
Climate (PCA1)	Tot VOCs C	−0.29	1.45	0.25	EPNs C	0.001	0.01	0.93
	Tot VOCs I	−0.50	5.21	0.04**	EPNs I	−0.53	6.37	0.02**
	Tot VOCs I-C	−0.25	1.02	0.32	EPNs I-C	−0.59	8.65	0.01**
	PCA VOCs	−0.25	1.08	0.31	EPNs es	−0.04	0.02	0.89
	H VOCs	0.24	0.97	0.34				

^†^r = coefficient of correlation; F_x,y_ = F value and associated degrees of freedom. Asterisks and degree symbols indicate significant (**, p < 0.05) or near significant (**°**, p ≤ 0.07) values.

### Phylogenetic escalation for VOCs production and EPNs recruitment

The radiation of *Festuca* along elevation gradients and across climatic niches carries phylogenetic signal that is expected under a Brownian motion model of trait evolution (K = 0.5, p = 0.04; K = 0.4, p = 0.06 respectively; Figs. S10 and S11, Table S5). Furthermore, our data was remarkably consistent with classic theory: we found phylogenetic escalation in the total amount and diversity of inducible VOCs (Table 1, Fig. 3). Specifically, after 7–9 divergence events from the split that separated the predominantly high altitudinal and latitudinal fine-leaved *Festuca* species from their broad-leaved congeners, between 13 and 16 million years ago, the plants’ inducible VOCs production and their diversity increased by an average of 4 folds when induced by herbivory. Additionally, we found a weak negative correlation (r = 0.43, estimate = −52.12, F_1,16_ = 3.79, p = 0.07) between branch length and the first axis of the principal component analysis of the VOCs, representing the axis of maximal scent variation across species (Fig. S10). This indicates that more ancestrally-diverged species tend to have similar chemical composition characterized by monoterpenes and low-weight hydrocarbon chains (VOCs 4, 5, 6, 9, 10, 13, 15), while more recently-diverged species tend to produce additional compounds, which, most importantly, included sesquiterpenes.

## Discussion

We tested three major predictions of classic plant defence theory regarding plant direct defences aboveground for the potential to explain a plants’ ability to recruit predators (i.e. indirect defences through tritrophic interactions) belowground. On one hand, findings showing that *Festuca* species adapted to high elevation cold and humid habitats had high predator recruitment after herbivore attack and chemical defence induction, provided evidence for the resource availability theory. On the other hand, we showed that increasingly-derived *Festuca* species were associated with highest emissions and diversity of volatile organic compounds (VOCs), thereby supporting the defence-escalation theory.

First, we found that root herbivory induced plants to be more attractive to soil-dwelling entomopathogenic nematodes (EPNs), in general, across 18 grass species of *Festuca*, when compared to undamaged plants. Aboveground herbivore-induced VOCs were shown to attract predatory arthropods (e.g., wasps and mites) in several dozens of systems and in a variety of habitats^2^. Later, root herbivores were also shown to induce root VOCs for attracting soil-dwelling EPNs^35^, but the effect of VOCs induction on predator recruitment were never tested across several plant species simultaneously. In summarizing work on chemically-mediated belowground tritrophic interactions, terpene-based compounds seem to play a major role in root defence^36^. For instance, corn plants genetically modified for enhanced *E*-β-caryophyllene production were more protected by EPNs against *Diabrotica virgifera* corn root larvae in the field^37^. Nonetheless, several other VOC classes can also induce attraction - as well as repulsion - of EPNs^35^. Because we found no direct correlation between total VOC production and EPN attraction, the unique identity of the VOC blends should be the main driver of EPN behaviour. Accordingly, previous work on *Festuca rubra* showed that EPN recruitment relies on the balance between the emissions of three attractants (α-curcumene, 1-undecene, nonadecatriene) and one deterrent (decane) compound^38^. Contrary to theory based on resource allocation within plants^39^, we observed a positive association between the total constitutive production of VOCs and their inducibility. Such positive association is not unprecedented^21,40–43^. For instance, positive correlations between constitutive and inducible defences were observed across >50 species of milkweeds (*Asclepias*) and across 21 wild cotton (*Gossypium*) species. Therefore, we consider our findings as a common pattern resulting from repeated evolution independently of the phylogenetic history, the geography of diversification, and of the cost of induction across species.

Second, our findings show that species growing at high elevation colder and more humid habitats, which are generally less productive^44^, harbour enhanced emission of root-herbivore induced VOC and better predatory recruitment power. These observations point to the prominent role of the resource availability hypothesis and refute the latitudinal/elevational gradient hypothesis. Our most strongly-supported climatic and soil predictors of induced VOCs production and EPN recruitment were related to temperature and humidity, and organic matter rich soils, respectively. Speciation into more climatically-stressed and resource-poor high elevations has thus been followed by a generalized adaptation to produce more attractive VOC blends. In the case of *Festuca*, we observed a relatively strong phylogenetic signal for elevation and climatic niche, indicating that more closely-related species tend to share more similar altitudinal zones (e.g., species of the *F. pratensis* clade in the phylogeny; Fig. S12)^45^. Nonetheless, we inferred several phylogenetically-independent transitions to high elevation niches across the phylogeny, as shown by the observation that representative alpine species span multiple clades in the phylogeny (Amphigenes: *F. pulchella*; Eskia-Dimorpha: *F. quadrifolia*; Exaratae: *F. violacea*; Festuca: *F. alpina*, *F. halleri*; Fig. 2, Figs. S12–14), thus having independently colonized the alpine zone regardless of phylogenetic history. Altogether, our results indicate that fescue radiation into alpine niches from different ancestors has resulted in optimal adaptation towards the production of highly attractive VOCs blends. This suggests that the resource-availability hypothesis is associated with a convergent evolutionary pattern. However, parallel to the plant adaptation perspective, we cannot exclude that insect adaptation may have also driven differences in VOC production along elevation. At low elevation, where plants and root herbivores co-occur frequently, selective pressures may have promoted insect adaptations enabling the control of plant VOC emissions. Along this line, the composition of oral secretions of adapted herbivores has been shown to limit VOC induction^46^. At the opposite end of the elevation gradient, a relaxation in plant–herbivore interactions may have hampered such insect adaptation process, thereby increasing VOC emissions at high elevation.

Third, we provide the first evidence of defence escalation for plant traits mediating tritrophic interactions between plants herbivores and predators. The continuous model of evolution we used indicates that defence escalation has occurred gradually via continuous character displacements in VOCs, but also according to subsequent speciation events; indeed we found strong correlation between VOCs (I, I-C, H) and the patristic distance and the number of intervening nodes from common ancestor (Table 1). *Festuca* ancestors likely originated in the lowland southern Palaearctic region and then diversified preferentially upwards into high mountains and also into high latitudinal Northern and Southern Hemisphere regions and colder climates^47–49^. Nonetheless, even when accounting for this directional radiation, we still found consistently stronger inducibility in species that inhabit higher altitudes. To date, other tests of the defence escalation hypothesis include interactions between plants and herbivores (i.e. direct defences). For instance previous work highlighted an increase in more toxic angular furanocoumarins in wild parsnip species^50^, an increase in tolerance (i. e., the ability to regrow) in milkweeds^51^ or an increase in alkaloid diversity in *Ficus* species^52^. While this pattern might apply to a wide range of defence traits, several studies failed to find correlation between phylogenetic branch lengths or speciation events and defence traits. For instance, the total amount of cardenolides decreases with increasing phylogenetic node numbers in milkweeds^51^. In the genus *Streptanthus*, while the total amount of glucosinolates varies independent of the phylogeny, the production of aliphatic glucosinolates increases over evolutionary time, while glucosinolate diversity declines^53^. Thus, our findings align with previous studies in that not all traits show consistent patterns of defence escalation. Indeed, while induction of VOCs in Alpine *Festuca* species correlates with phylogenetic branch length, the constitutive production of VOCs varies independently of the phylogeny.

Altogether, we have shown that for high-elevation *Festuca* species, herbivores induce consistently more and more diverse VOC emissions and attract more EPNs than their low-elevation counterparts. Moreover, our results suggest that higher EPN recruitment in harsh conditions relies on specific VOC profiles rather than on total amount of VOC. This is in line with the assumption that the cost of herbivory depends on the plant’s opportunities to acquire resources. In this case, the short growing season of alpine environments makes the period of resource acquisition brief and intermittent during the growing season. While high-elevation soils contain lower diversity and abundance of arthropods and predators^6^ we cannot completely exclude moderate amounts of root herbivory by different guilds, and thus we expect selection of the induction of optimally-attractive VOC blends upon unexpected herbivore attack. Finally, with high altitude conditions, we might also expect pleiotropy of plant defence traits, in which the production of specific secondary metabolites can ameliorate plant fitness in harsh environments. Indeed, a recent review has indicated that VOC emission rates for phototrophs in extreme environments are frequently higher than that of organisms from less stressful environments^54^. Therefore, the reason for widespread VOC emissions from different extreme environments and its association with herbivore pressure and phylogenetic history deserves further attention, as these compounds may have important roles in stress resistance and adaptation to extreme habitats.

## Material and Methods

### Study system

With more than 500 species described, the genus *Festuca* represents a major evolutionary line of the tribe Poeae. These tufted grasses are herbaceous perennials with a height range of 10–200 cm and a cosmopolitan distribution, growing on every continent except Antarctica^47,55^. Within the entire European Alpine range, there are about 50–70 species depending on their classification^56^. From this range, we collected different taxa at 31 sites (Fig. S1, Table S1), ultimately selecting 18 confirmed species with which we were able to perform belowground olfactometer behavioral assays (see below). The plants were collected from pristine sites that represent the optimal niche of each species; www.infoflora.ch) (Fig. S11). At each site, we excavated 20–30 individuals with their own soil and potted them in plastic pots (20 cm diameter, 25 cm high) by completing the native soil with standard soil mixture composed of 2/3 regular potting soil (5 parts compost, 4 parts peat substitute based on wood fiber and 1 part topsoil, pH in water = 7.45, conductivity = 2 mS cm^−1^, Ricoter, Aarberg, Switzerland) and 1/3 sand in garden plastic pots (25 cm diameter, 30 cm high) in an outdoor common garden at the Botanical Garden of Neuchâtel, Switzerland. For phylogenetic reconstruction of the *Festuca* species see details in supplementary methods, Tables S1–S2, and Figs. S12–S14.

At each collection site, we also collected soil samples to characterize edaphic variables associated with each species. We sampled about 1 Kg of soil from 10–30 soil cores (5 cm wide and 20 cm deep). Soils samples were homogenized, dried at 40 °C for 48 hours, sieved at 2 mm, and ground using agate mortars for subsequent physicochemical analyses. Specifically, we measured (1) relative humidity (HR) by weighting soils before and after drying at 105 °C for 3 days; (2) organic matter content (OR) through loss on ignition by weighting before and after burning 10 gr of soil at 450 °C for 2 hrs; (3) pH in 2.5 soil volume of deionized water; (4) cation exchange capacity (CEC) following the cobaltihexamine chloride method; (5) carbon to nitrogen ratio (CN) using an elemental analyser (FLASH2000, Thermo Fisher Scientific, Waltham, Massachusetts, United States); (6) total carbonates (CaCO3) using a calcimeter (Bernard type), and (7) bioavailable phosphorous (P_bio) using the Kjeldahl digestion method^57^. Additionally, for each species we extracted ecological indicator values for soil fertility (Soil_N) based on Landolt *et al*.^58^. To measure EPNs infectivity potential of each site we ran an infectivity test shortly after soil collection (maximum one week) to avoid loss of potential EPNs present in soil. Three soil subsamples (200 g) were added with five larvae of the greater wax moth (*Galleria mellonella*) and stored in the dark^59^. After six days we checked for dead larvae, which were placed in White traps for nine days and monitored for EPNs emergence. The experiment was run twice on the same soils to corroborate initial results. The average values of each soil variable are summarized in Table S3.

### Root herbivores and predatory nematodes

In order to study *Festuca* interspecific variation in belowground tritrophic interactions along environmental gradients, we used the dietary generalist herbivore, *Melolontha melolontha* (Coleoptera: Scarabaeidae). The larvae develop in soils by feeding on roots for up to three years, causing severe damage to plants, particularly to Poaceae in low-elevation grasslands, where herbivore population densities can reach 100 individuals/m^2^, but the beetle larvae practically disappear in the Alpine zone^60^. *M. melolontha* larvae were collected in pastures around Switzerland and stored at 10 °C in potting soil. Although *M. melolontha* is generally found between 300 and 600 m above sea level (asl), Scarabaeidae root herbivores can easily cause damages in mountainous zones^61^. While we used a single herbivore species in order to compare herbivore effects on different plant species, we assumed that the generalist diet of *M. melolontha* enables to extrapolate herbivore effects of closely-related Scarabaeidae larva damaging mountainous zones. As soil predators, we used *Heterorhabditis megidis* (Rhabditida: Heterorhabditidae) entomopathogenic nematodes (EPNs) (Andermatt Biocontrol, Andermatt, Switzerland). EPNs mode of action can be summarized in three steps: i) penetration into the arthropod host by the third instar resistant stage juveniles, ii) release of symbiotic bacteria in the hemolymph of the host and iii) EPNs feeding on the newly-formed bacterial soup for 2–3 generations inside the host^62^. Species of EPNs in the genera *Steinernema* and *Heterorhabditis*, including *H. megidis*, are found in most habitats, including the Swiss Alps. In a previous survey of nematodes along elevation gradients, we collected *H. megidis* up to 2000 m asl, and we observed that EPNs, in general, range from 500 to 2750 m. asl^63^. Most EPNs actively seek their hosts in the soil and, while CO_2_ can serve as a general cue for host searching, more specific plant-associated volatile compounds have been shown to inform EPNs of the presence of a root insect herbivore in the surroundings^4,64^. *H. megidis* relies on host-plant cues for several systems such as coniferous plants, grasses and other angiosperms^36^. Additionally, nematodes belonging to *Heterorhabditis* genera cause relatively fast larval mortality (i.e., after 7 days) for different Scarabaeidae species^65^.

### Belowground olfactometer bioassays

After a period of about 10 months (from summer 2016 to spring 2017), including an overwintering period of about 6 months in which all aboveground parts of the plants were cut to the ground, 5 plants per species were randomly selected from the initial pool and divided into two equally-sized tufts for performing olfactometer bioassays. For three species with wide distribution ranges (*F. heterophylla, F. ovina*, *F. rubra*), we doubled the sample size in order to include two distinct collection sites per species. Individual tufts of each species were separated in half to obtain unique clonal pairs of plants, which were potted in custom-made two-arm belowground olfactometer pots as described in Kergunteuil *et al*.^6^, one pair of clones per olfactometer arm and filled with the same soil mixture as previously described. One month later, one plant per olfactometer was infested with three *M. melolontha* L2 larvae that were previously starved for three days. Two days after the plant infestation, the central pieces of the olfactometers were filled with 10% moist sand (Neogard, Gontenschwil, Switzerland) and assembled with each pair of plants. Three days after the initial damage, 2000 six-day-old *H. megidis* EPNs were released in the central chamber of the olfactometers and allowed to move toward either uninfested or infested plants for 24 hours. The behavioural choices of nematodes were recorded by counting the individuals collected in the arms connected to ‘uninfested’ and ‘infested’ treatments after the Baermann funnel extraction^4^.

### Chemical analyses of VOCs

Immediately after the olfactometer bioassays described above, all the plants were removed from the pots, and roots were carefully washed and ground to a fine powder in liquid nitrogen. The root material was then stored at −80 °C before chemical analyses of volatile organic compounds. All analyses were done with a gas chromatograph (GC) (Agilent 7890A) coupled with a mass spectrometer detector (MSD) (Agilent 5975C). The samples were prepared and introduced into the GC with the use of robotic multipurpose samplers (MPS, Gerstel GmBH). The compounds were separated on Agilent HP-5MS columns (30 m length × 0.25 mm i.d., and 0.25 µm film thickness). In all cases, the MSD transfer line temperature was set at 280 °C and the ion source and quadrupole temperatures were set at 230 °C and 150 °C, respectively. The electron impact (EI) mode was used with a scanning over the mass range of 33–250 m/z. The chemical compounds were trapped through the use of solid-phase microextraction (SPME) headspace technique. The samples were incubated in a 20 ml glass vial for 3 minutes at 35  °C before inserting a 100 µm polydimethylsiloxane (PDMS) coated fiber (Supelco, Bellefonte, USA) into the headspace for 20 minutes. Afterward, the compounds were thermally desorbed from the fiber for 210 seconds (splitless mode, 250 °C, 6.5 psi pressure, 210 mL/min purge flow, helium carrier gas) before injection onto the GC column. The initial column temperature of 50 °C was held for 1 min, then it was ramped at 6 °C/min until 250 °C (hold time 1 min), and finally a 3 min post run at 260 °C. The helium flow rate was 0.9 mL/min (constant flow mode). The analysis of root-emitted volatiles was based on 26 compounds that were not detected in blank samples. While 3 volatiles were identified using pure standards, the additional volatiles were tentatively identified by comparing the mass spectra with the NIST05 mass spectra library (Table S4). The peak area for each detected compound was divided by the peak area of the internal standard (Tetralin®, Sigma-Aldrich, St. Gallen, Switzerland), and the root emissions of volatiles were finally given as tetralin equivalent nanograms of compound released by gram of root biomass collected^66^. For subsequent statistical analyses, we calculated the total amount of VOCs produced per species, and the Shannon diversity index H (function *diversity* from the package vegan^67^). We did not include the number of individual peaks (i.e. peak richness), since it is highly correlated with both the total amount (t_1,158_ = 2.92, p < 0.001), and the Shannon diversity (t_1,158_ = 6.30, p < 0.001).

### Statistical analyses

#### Effect of herbivory on root biomass, nematode recruitment and VOCs production

To address the impact of the herbivore treatment, we first calculated standardized effect sizes for root biomass and EPNs recruited between herbivore-infested plants minus the control plants using Hedge’s g statistics (function *cohen.d* in package effsize in R^68^). We next used Bayesian phylogenetic mixed models (BPMMs), as implemented in the R package MCMCglmm^69^, to test the effect of the herbivore treatment on average root biomass, EPNs recruitment, total and all individual VOCs emitted across all species, while taking into account phylogenetic relationships among *Festuca* species. Differences of VOCs production across species and herbivore treatment were also assessed with a permutational multivariate analysis of variance (PERMANOVA) based on Bray-Curtis distance matrices (function *adonis* from the package vegan^67^), and average species and treatment differences were visualized using non-metric multidimensional scaling (NMDS) (function *metaMDS* in vegan) (Fig. S3). All VOCs data were log-transformed to normalize their distribution (function *decostand* in vegan). To test for potential negative correlations between constitutive VOCs (species means) and their inducibility (i.e., the difference in mean VOCs values for each species between control and damaged plants), we conducted tests to control for potential spurious correlations^70^ in Matlab (version 7.5.0.342 – R2007b, MathWorks Inc., USA). We also performed phylogenetically controlled analyses to tests for correlations between mean constitutive VOCs levels and their inducibility.

#### Phylogenetic signals and tests of the defence escalation hypothesis

We first estimated the phylogenetic signal (Blomberg’s K) for all individual VOCs, their sum and their Shannon diversity, and the climatic niche (PCA1) using species-level averages with the function *phylosig* of the package *phytools* in R^71^. A K value of 1 indicates that trait values are consistent with the tree topology and a random walk model (i.e., trait similarity is directly proportional to the extent of shared evolutionary history). A K value close to 0 indicates no influence of shared ancestry on trait values (i.e., phylogenetic independence). When K > 1, the traits have more phylogenetic signal than expected from a random walk model on the given phylogeny^45^. Next, to test for the defence escalation hypothesis, we calculated the patristic distance from root to tip (i.e. the number of apomorphic step changes separating two taxa on a cladogram), for determining the amount of divergence of each species from their common ancestor. We then performed correlation analyses between the patristic distance and the constitutive, induced and inducibility of VOCs and nematode recruited trait values of the 18 species of *Festuca*. We also regressed the first axis of a principal component analysis (PCA) of all VOCs (function *dudi.pca*, packages ade4^72^) against patristic distance. Finally, in addition to using branch length, we also regressed traits values against the number of intervening nodes from root to tip for each species (e.g. as in Agrawal and Fishbein^51^), although the two measures are highly correlated; r = 0.88, t_1,16_ = 6.89, p < 0.001).

#### Climate effect on VOCs production and nematode recruitment

To measure the effect of climate on constitutive, induced and inducibility of VOCs production and EPN attraction, we first constructed the climatic niche optima for each species based on occurrence data, and the first axis of the ordination of the climatic variables (Figs. S6 and S7). We then regressed this first PCA axis with each response variable using phylogenetic corrected analyses (PGLS) (function *pgls*, package caper^73^). In all cases, to reduce Type I errors, we optimized branch length thorough maximum likelihood^74^. We next dissected the importance of each individual climatic variable by running a multivariate PGLS regression model.

#### Soil effect on VOCs production and nematode recruitment

To address potential correlations between the soil physico-chemical variables and the whole VOCs matrix we mapped the soil variables (Table S3, Fig. S8) on the VOCs ordination based on a distance-based redundancy analysis (dbRDA) (function *cca* in the package vegan). We performed the analysis using both Euclidean and Bray-Curtis distances, and obtained very similar results, and thus used the Euclidean distance results to show. Finally, we performed a multivariate regression analysis for testing the effect of all soil variables on EPNs attraction.

## Supplementary information

Supplementary information.

## Data Availability

Data underlying this article can be accessed on Dryad Digital Repository at http://dx.doi.org/XXX/doi.goes.here, and used under the Creative Commons Attribution licence.
