# Supplementary material for: Tritrophic interactions follow phylogenetic escalation and climatic adaptation
Source: Sci Rep. 2020 Feb 7;10:2074. doi: 10.1038/s41598-020-59068-2 (PMC7005781; doi:10.1038/s41598-020-59068-2)
Supplement: Supplementary file 1 — Supplementary information. [file 41598_2020_59068_MOESM1_ESM.docx]

**Supplementary material for:**

**Tritrophic interactions follow phylogenetic escalation and climatic adaptation**

Alan Kergunteuil, Laureline Humair, Anne-Laure Maire, María Fernanda Moreno, Adrienne Godschalx, Pilar Catalán, Sergio Rasmann

**Supplementary methods**

***Phylogenetic reconstruction***

We estimated the phylogeny of the 18 *Festuca* species by reconstructing their phylogenetic position within a larger genus-wide evolutionary framework ^1,2^, specifically by including an additional 27 species representing the main broad-leaved and fine-leaved lineages of *Festuca* (Loliinae, Pooideae) occurring in the Alps and other Holarctic ranges (Table S2, Figure S1). *Poa alpina* (Poinae, Pooideae), *Secale cereale* (Triticeae) and *Brachypodium distachyon* (Brachypodieae) were used as outgroups (Table S2). DNA sequence data of the nuclear ribosomal ITS (ITS1-5.8S-ITS2) region and the plastid trnTL and trnLF regions for all species but four were retrieved from our previous works (Table S2) ^1,2^. New nuclear and plastid DNA sequences were generated for *F. acuminata, F. brevipila* and *F. filiformis* following the DNA amplification and sequencing procedures indicated in Inda et al. ^1^ and for *F. nigrescens* using a genome skimming approach. It consisted of high quality DNA isolation using the QIAGEN kit followed by shallow genome sequencing in a CNAG (Centro Nacional de Análisis Genómicos) Illumina HiSeq4000 platform that rendered 23 million of 2x100bp paired-end (PE) reads. The whole *F. nigrescens* nuclear ribosomal DNA cistron and plastome were assembled from curated PE reads with Geneious v. R11 using the *Brachypodium distachyon* rDNA cistron (IBI 2010) and the *Festuca ovina* plastome ^3^ as references. Nuclear ITS, plastid trnTL and trnLF sequences of *F. nigrescens* were retrieved from the respective rDNA cistron and plastome assemblies and were aligned with the respective *F. acuminata, F. brevipila*, *F. filiformis* and remaining *Festuca* and outgroup sequences using the MAFFT algorithm implemented in Geneious R11. Phylogenetic analyses of nuclear ITS and plastid trnTL-trnLF sequences were performed separately using Maximum Likelihood and the Bayesian approaches and employing *Brachypodium* to root the trees (Figures S3, S4). After phylogenetic reconstruction, the tree was pruned using the *drop.tip* function of the ape package in R ^4^. While both the ML IQTREE and Bayesian BEAST trees recovered overall highly congruent topologies, we found an incongruent phylogenetic position of *F. nigrescens*, nesting in the Aulaxyper clade in the nuclear tree (Figure S3), and in the Festuca clade in the plastid trees (Figure S5), which might indicate an hybridogenous origin of this sample. *F. nigrescens* has been traditionally classified within the Aulaxyper group ^5^ and is considered a purported allohexaploid species ^6^. Our nuclear and plastid phylogenetic data suggest a cross between paternal *F. rubra*-type and maternal *F. ovina*-type lineages in the origin of this sample that shows typical phenotypic and ITS features of the *F. rubra* group but a captured *F. ovina*-like chloroplast. Due to the overall Aulaxyper phenotype characteristics of *F. nigrescens*, we chose the ITS-based tree as the most accurate topology to investigate the evolution of herbivory-related traits along the phylogeny of the studied *Festuca* species (Figure 1).

**Supplementary Tables**

**Table S1**. Collection sites for 18 species of *Festuca* growing in the Swiss Alps. Shown are the collection date, the taxon name, the habitat range based on Aeschimann et al. ^7^, Swiss grid (CH1903 / LV03GPS) GPS coordinates, and elevation of each site.

| Site | Collection date | Taxon | Habitat | X | Y | Elevation(m) |
| --- | --- | --- | --- | --- | --- | --- |
| 1 | 14.05.2016 | Festuca acuminata Gaudin | mountain-alpine | 703339 | 149708 | 1015 |
| 26 | 21.07.2016 | Festuca alpina Suter | subalpine-alpine | 615963 | 145918 | 2240 |
| 31 | 23.08.2016 | Festuca alpina Suter | subalpine | 747191 | 168169 | 2780 |
| 19 | 19.07.2016 | Festuca amethystina L. | colline-mountain | 549431 | 200759 | 720 |
| 16 | 06.07.2016 | Festuca arundinacea Schreb. s.l. | colline-mountain | 568165 | 116343 | 435 |
| 3 | 14.05.2016 | Festuca brevipila R. Tracey | colline-subalpine | 705109 | 148322 | 776 |
| 6 | 14.05.2016 | Festuca brevipila R. Tracey | colline-subalpine | 704077 | 148723 | 776 |
| 8 | 25.05.2016 | Festuca brevipila R. Tracey | colline-subalpine | 568041 | 116453 | 443 |
| 5 | 14.05.2016 | Festuca filiformis Pourr. | colline-alpine | 705109 | 148322 | 776 |
| 20 | 19.07.2016 | Festuca gigantea (L.) Vill. | colline-mountain | 549067 | 200826 | 620 |
| 15 | 06.07.2016 | Festuca heterophylla Lam. | colline-mountain | 561919 | 206629 | 730 |
| 11 | 20.06.2016 | Festuca laevigata Gaudin s.l. | colline-alpine | 618914 | 129440 | 1050 |
| 14 | 04.07.2016 | Festuca laevigata Gaudin s.l. | colline-alpine | 571590 | 219980 | 1440 |
| 18 | 12.07.2016 | Festuca laevigata Gaudin s.l. | colline-alpine | 631693 | 125603 | 1530 |
| 27 | 21.07.2016 | Festuca nigrescens Lam. | colline-alpine | 616233 | 145452 | 1896 |
| 10 | 22.06.2016 | Festuca ovina aggr. | colline-mountain | 564542 | 207947 | 630 |
| 13 | 21.06.2016 | Festuca ovina aggr. | colline-mountain | 550918 | 198362 | 739 |
| 25 | 21.07.2016 | Festuca ovina aggr. | colline-mountain | 615958 | 146039 | 2150 |
| 30 | 03.08.2016 | Festuca paniculata (L.) Schinz & Thell. | subalpine | 725142 | 125660 | 2100 |
| 12 | 21.06.2016 | Festuca pratensis Huds. s.l. | colline-subalpine | 551620 | 197966 | 573 |
| 17 | 12.07.2016 | Festuca pratensis Huds. s.str. | colline-subalpine | 630348 | 124407 | 1640 |
| 22 | 20.07.2016 | Festuca pulchella Schrad. s.str. | subalpine-alpin | 613930 | 142383 | 2164 |
| 24 | 20.07.2016 | Festuca quadriflora Honck. | subalpine-alpine | 614234 | 142463 | 2103 |
| 2 | 13.05.2016 | Festuca rubra L. s.str. | colline-alpine | 568417 | 115917 | 533 |
| 4 | 14.05.2016 | Festuca rubra L. s.str. | colline-alpine | 705109 | 148322 | 776 |
| 23 | 20.07.2016 | Festuca rubra L. s.str. | colline-alpine | 613930 | 142383 | 2164 |
| 7 | 24.05.2016 | Festuca valesiaca Gaudin | colline-subalpine | 610222 | 128085 | 563 |
| 21 | 20.07.2016 | Festuca violacea Gaudin | subalpine-alpin | 613905 | 142313 | 2170 |
| 29 | 27.07.2016 | Festuca violacea Gaudin | subalpine-alpine | 610749 | 105716 | 2400 |

**Table S2**. *Festuca* and outgroup taxa included in the phylogenetic study. Source of samples and Genbank accession codes of the nuclear and plastid DNA sequences used in phylogenetic analysis. Sample records in bold correspond to newly deposited sequences obtained in this study. The nomenclatural, supertribal, tribal, generic and sub-generic classification and phylogenetic adscriptions of *Festuca* taxa and lineages follow those proposed by Inda *et al. ^1^*, Minaya *et al*. ^2^ and the current study. Voucher information (collector, collector number, herbarium, herbarium number), DNA bank or sequence bank information is indicated for each sample. Herbaria and DNA bank codes: JACA-Instituto Pirenaico de Ecologia, CSIC, Spain; LEB-Universidad de Leon, Spain; UAM-Universidad Autonoma de Madrid, Spain; US- United States National herbarium, Smithsonian, USA; UZ-Universidad de Zaragoza, Spain. UniNE- Botanical Garden of Neuchâtel.

|  | | | | | | |
| --- | --- | --- | --- | --- | --- | --- |
| **Taxon** | **Source** |  | **GenBank accession** | | | |
|  |  |  | **ITS** | ***trn*LF** | | ***trn*TL** |
| **Poeae R. Br.** | | | | | | |
| **Loliinae Dumort.** | | | | | | |
| **Broad-leaved Loliinae** | | | | | | |
| **Lojaconoa clade** | | | | | | |
| *Festuca coerulescens* Desf. | Spain: Cadiz: Jerez de la Frontera; Catalan P. & al., UZ-91.2000 |  | AF538363 | AF533051 | | EF585027 |
| **Drymanthele clade** | | | | | | |
| *Festuca altissima* All. | France: Pyrenees: Aspe; Catalan P. UZ |  | AF303411 | AF478505 | | EF585003 |
| **Leucopoa – Amphigenes clade** | | | | | | |
| *Festuca pulchella* Schrad. | Switzerland: Bern; Muller J. 7807 UZ |  | AF519980 | AF519985 | | EF585086 |
| *Festuca kingii* (S. Watson) Cassidy | USA: Colorado: Boulder Co: Flat Irons; Catalan P. UZ-1.93 |  | AF303410 | AY099004 | | EF585058 |
| **Subbulbosae clade** | | | | | | |
| *Festuca paniculata* (L.) Schinz | France: Mont Aigoual; Cebolla C & Rivas-Ponce MA, UAM |  | AF303407 | AF533046 | | EF585077 |
| **Schedonorus clade** | | | | | | |
| *Festuca arundinacea* Schreb. subsp. *arundinacea* | Spain: Lugo: Láncara: Santa Bárbara; López Rodríguez J.A. 1081, UZ |  | AF519976 | AY098995 | | DQ367405 |
| *Festuca gigantea* (L.) Vill. | Spain: Navarra: Arce; Aizpuru I & Catalan P, UZ |  | AF303416 | AF533043 | | EF379003 |
| *Festuca pratensis* (Huds.) P. Beauv. | England: Wilshire: Calne; Stace C.A., UZ DNA bank |  | AF303421 | AF478503 | | EF379007 |
| **Fine-leaved Loliinae** | | | | | | |
| **Eskia–Dimorpha clade** | | | | | | |
| ***Festuca acuminata* Gaudin** | **Switzerland: Ticino: Faido: ; Suhner M. & al., UniNe** |  | **xxxxxx** | **xxxxxx** | | **xxxxxx** |
| *Festuca burnatii* St.-Yves | Spain: Cantabria: Picos de Europa; Catalan P. &Torrecilla P., UZ-44.2001 |  | AY099007 | AY099002 | | EF585019 |
| *Festuca dimorpha* Guss. | France: Alpes de Haute-Provence Col des Champs; Korneck D. s.n, Herb. Muller J. 10969, UZ DNA bank |  | AF519982 | AF519987 | | EF585032 |
| *Festuca eskia* Ramond ex DC. | Spain: Huesca: Pyrenees: Benasque; Catalan P., Mirones V. UZ |  | AF303412 | AF478508 | | EF585040 |
| *Festuca gautieri* (Hack.) K.Richt. | Spain: Girona: Pyrenees: Nuria; Catalan P., Mirones V. UZ |  | AF303414 | AF478507 | | EF585044 |
| *Festuca pumila* Chaix | Slovak Republic: Veliki Stador. Gutermann 38499, UZ 4.08 |  | KY368817* | KY368868* | | KY368918* |
| *Festuca quadriflora* Honck | France: Pyrénées: Col de Baroude; Catalán P. UZ-2000 |  | AF303413 | AF478506 | | EF585089 |
| **Exaratae clade** | | | | | | |
| *Festuca amethystina* L. | Germany: Bayern; Muller J. 6966, UZ DNA bank |  | EF584919 | EF592950 | | EF585004 |
| *Festuca capillifolia* Dufour | Spain: Jaen: Cazorla; Cebollada , Rivas-Ponce M.A., UAM |  | AF303419 | AF478511 | | EF585022 |
| *Festuca norica* (Hack.) K. Richt. | Italy: Trentino-Alto Adige; Muller J. 8422, UZ |  | EF584955 | EF592987 | | EF585072 |
| *Festuca violacea* Gaudin | Switzerland: Bern; Muller J. 7907, UZ DNA bank |  | EF584979 | EF593012 | | EF585113 |
| **Festuca clade** | | | | | | |
| *Festuca alpina* Suter | Spain: Huesca: Pyrenees: Vallibierna; Catalan P., UZ-2002 |  | AF303415 | AF478522 | | EF585001 |
|  |  |  |  |  | |  |
| ***Festuca brevipila* R. Tracey** | Switzerland: ; Suhner et al., UniNe |  | **xxxxxx** | **xxxxxx** | | **xxxxxx** |
| *Festuca brevissima* Jurtzev | USA: Alaska: Denali Borough: Alaska Range; Soreng R.J.6021 US |  | EF584928 | EF592961 | | EF585018 |
| ***Festuca filiformis* Pourr.** | **Switzerland: Ticino: Faido; Suhner M. & al., UniNE** |  | **xxxxxx** | **xxxxxx** | | **xxxxxx** |
| *Festuca glacialis* Miègev. ex Anon | Spain: Huesca: Pyrenees: Cotiella; Catalan P., UZ-2002 |  | AF303428 | AF478523 | | EF585045 |
| *Festuca halleri* All. | Switzerland: Valais; Muller J. 8032, UZ |  | EF584942 | EF592975 | | EF585047 |
| *Festuca hyperborea* Holmen ex Fred. | Canada, Northwest Territories, Prince Patrick Island; Gillespie L.J., Consaul L.L. 6893, UZ DNA bank |  | EF584946 | EF592978 | | EF585050 |
| *Festuca hystrix* Boiss. | Spain: Almerıa: Sierra de Gador; Catalan P. & al., UZ- 31.2000 |  | AF478480 | AF478520 | | EF585051 |
| *Festuca indigesta* Boiss. | Spain: Granada: Sierra Nevada; Catalan P. & al., UZ-43.2000 |  | AF303426 | AF478519 | | EF585054 |
| *Festuca intercedens* (Hack.) Lüdi ex Bech. | Switzerland: Valais: Muller J. 7987, UZ DNA bank |  | EF584948 | EF592979 | | EF585055 |
| *Festuca laevigata* Gaudin | Italy: Lombardia. Muller J. 8267, UZ DNA bank |  | EF584950 | EF592981 | | EF585059 |
| *Festuca longiauriculata* Fuerte, Ortúnez et Ferrero | Spain: Almería: Sierra de los Filabres: Calar Alto; Catalán P. & al., UZ- 59.2000 |  | AF478479 | AF478518 | | EF585062 |
| *Festuca ovina* L. | Germany: Thuringen: Saale-Holzland-Kreis; Muller J. 6879, UZ DNA bank |  | AF532959 | AF533063 | | EF585076 |
| *Festuca panciciana* K. Richt. | Bosnia-Herzegovina: Troglav: Sajkovacko zdrlo. Gutermann et al., UZ |  | JQ972951 | JQ972974 | | JQ973015 |
| *Festuca valesiaca* Schleich. ex Gaudin | Germany: Thuringen; Muller J. 6939, UZ DNA bank |  | EF584978 | EF593011 | | EF585112 |
| **Aulaxyper clade** | | | | | | |
| *Festuca agustinii* Linding. | Spain: Canarias: Tenerife: Anaga: Bailadero; A.Santos, UZ DNA bank |  | AY099005 | AY099003 | | EF584999 |
| *Festuca ampla* Hack. | Spain: Cadiz: Grazalema; Lopez Rodrıguez J.A., 1326, UZ |  | EF584921 | EF592953 | | EF585007 |
| *Festuca heterophylla* Lam. | France: Pyrénées-Orientales Forêt de Boucheville; Montserrat P & al. s.n. JACA-152778 |  | EF584944 | - | | EF585049 |
| *Festuca iberica* (Hack.) K. Richt. | Spain: Granada: Sierra Nevada: Borreguiles de S. Juan; Catalan P. & al., UZ- 77.2000 |  | AY118087 | AF478516 | | EF585052 |
| *Festuca jubata* Lowe | Portugal: Madeira: Pico das Torres; Sequeira M & Catalan P, UZ DNA bank |  | EF584949 | EF592980 | | EF585056 |
| *Festuca juncifolia* Chaub. | Spain: Lugo Viveiro: Brieiro: Arenales de Area; López-Rodríguez J.A. 1366, UZ |  | AF478478 | AF478515 | | EF585057 |
| ***Festuca nigrescens* Lam.** | **Switzerland: Kandersteg: Bern; Suhner M. & Maire A.-L., UniNE** |  | **xxxxxx** | **xxxxxx** | | **xxxxxx** |
| *Festuca petraea* Guthnick | Portugal: Azores: St. Maria, Maia; Sequeira M. 4393, UZ DNA bank |  | EF584962 | EF592994 | | EF585081 |
| *Festuca pruinosa* (Hack.) Patzke | Spain: Pontevedra: Cangas de Morrazo, Donon; Sauquillo E & Pimentel M, UZ. |  | EF584963 | EF592995 | | EF585083 |
| *Festuca rivularis* Boiss. | Spain: Granada: Sierra Nevada: Borreguiles de S. Juan; Catalan P. & al., UZ-78.2000 |  | AF478475 | AF478512 | | EF585093 |
| *Festuca rubra* L. | Finland: A.Kosonen JACA JA-474496 |  | EF584968 | EF593001 | | EF585097 |
| **Outgroups** | | | | | | |
| **Brachypodieae** Harz | | | | | | |
| *Brachypodium distachyon* (L.) P.Beauv. | Spain: Caceres. UZ 28.07 | | AF303399 | AF478500 | | DQ336855 |
| **Triticeae** Dumort. | | | | | | |
| *Secale cereale* L. | Genbank | | DQ981410 | AB732940 | JQ973001 | |
| **Poeae** R.Br | | | | | | |
| *Poa alpina* L. | Andorra: Ordino. Llamas, Acedo C & Alonso A, 62, LEB | | EU792390 | AY504635 | | DQ353986 |

**Table S3.** Soil traits. HR = relative humidity (%); OM = organic matter based on loss of ignition experiment; pH = water-based pH; CEC = cation exchange capacity; CN: carbon to nitrogen ratio; CaCO_3_ = amount of calcium carbonate; P_bio = bioavailable phosphorous; Infectivity = entomopathogenic nematodes infectivity rates based on the *Galleria mellonella* bioassay.

| Species | HR | OM | pH | CEC | CN | CaCO3 | P_bio | Infectivity | Soi_N |
| --- | --- | --- | --- | --- | --- | --- | --- | --- | --- |
| *Festuca_acuminata* | 3.19 | 20.71 | 5.70 | 16.40 | 19.46 | 0.00 | 0.03 | 0.03 | 2.00 |
| *Festuca_alpina* | 3.60 | 21.89 | 5.37 | 16.00 | 16.08 | 0.00 | 0.02 | 0.00 | 1.00 |
| *Festuca_altissima* | 2.50 | 11.41 | 6.60 | 22.50 | 16.95 | 0.24 | 0.01 | 0.03 | 3.00 |
| *Festuca_amethystina* | 10.54 | 23.64 | 7.42 | 44.10 | 17.90 | 21.26 | 0.02 | 0.03 | 2.00 |
| *Festuca_arundinacea* | 2.47 | 10.56 | 7.30 | 21.00 | 11.32 | 30.07 | 0.03 | 0.37 | 4.00 |
| *Festuca_brevipila* | 1.84 | 10.08 | 6.23 | 14.03 | 12.90 | 6.60 | 0.01 | 0.12 | 2.00 |
| *Festuca_filiformis* | 1.90 | 11.28 | 5.65 | 10.90 | 13.02 | 0.00 | 0.01 | 0.10 | 2.00 |
| *Festuca_gigantea* | 5.00 | 20.00 | 7.07 | 44.00 | 15.68 | 3.70 | 0.01 | 0.10 | 3.00 |
| *Festuca_halleri* | 0.68 | 4.38 | 6.84 | 6.20 | 18.89 | 0.17 | 0.01 | 0.17 | 2.00 |
| *Festuca_heterophylla* | 4.34 | 17.31 | 6.57 | 36.10 | 17.02 | 0.00 | 0.01 | 0.07 | 2.00 |
| *Festuca_laevigata* | 3.84 | 16.14 | 6.71 | 25.27 | 12.90 | 0.41 | 0.01 | 0.09 | 2.00 |
| *Festuca_nigrescens* | 10.17 | 30.23 | 6.14 | 37.30 |  | 0.00 | 0.04 | 0.07 | 3.00 |
| *Festuca_ovina* | 5.91 | 23.20 | 7.13 | 42.13 | 11.98 | 22.10 | 0.02 | 0.18 | 2.00 |
| *Festuca_paniculata* | 5.46 | 32.97 | 4.27 | 9.20 | 15.11 | 0.20 | 0.01 | 0.10 | 3.00 |
| *Festuca_pratensis* | 2.90 | 11.55 | 6.75 | 22.50 | 12.87 | 2.26 | 0.01 | 0.07 | 4.00 |
| *Festuca_pulchella* | 3.84 | 15.62 | 6.85 | 29.70 | 11.70 | 0.00 | 0.01 | 0.17 | 3.00 |
| *Festuca_quadriflora* | 14.22 | 36.10 | 6.87 | 48.50 | 11.93 | 1.25 | 0.08 | 0.10 | 2.00 |
| *Festuca_rubra* | 2.50 | 11.73 | 6.60 | 19.53 | 11.75 | 2.05 | 0.01 | 0.19 | 3.00 |
| *Festuca_valesiaca* | 1.00 | 5.69 | 7.51 | 11.30 | 11.27 | 44.05 | 0.01 | 0.00 | 2.00 |
| *Festuca_violacea* | 4.46 | 17.08 | 6.87 | 23.95 | 16.41 | 0.76 | 0.02 | 0.10 | 3.00 |

**Table S4.** MCMCglmm table for testing the effect of herbivore feeding on the production of all individual VOCs, total amount of VOCs and diversity of VOCs, across all 18 species of *Festuca* while taking into account the phylogenetic relationship between species.

| VOCs | Names | post.mean | l-95% c.i. | u-95% c.i. | eff.samp | pMCMC |  |
| --- | --- | --- | --- | --- | --- | --- | --- |
| VOC1 | 1R-a-Pinene | -2.1132 | -6.5111 | 2.6536 | 1000 | 0.39 |  |
| VOC2 | p-Menthene | 1.9298 | 1.1279 | 5.5297 | 1059 | 0.24 |  |
| VOC3 | b-Pinene | 6.74 | 3.262 | 18.089 | 1000 | 0.234 |  |
| VOC4 | 1-Octen-3-ol | 4.691 | 11.776 | 19.506 | 1000 | 0.56 |  |
| VOC5 | Octanone | 13.1 | -31.02 | 52.12 | 1000 | 0.512 |  |
| VOC6 | 3-Octanol | -3.284 | 14.59 | 9.324 | 1048 | 0.604 |  |
| VOC7 | Eucalyptol | 0.8807 | 2.4633 | 3.9381 | 1000 | 0.594 |  |
| VOC8 | 1-Undecene | 0.119 | -7.048 | 7.764 | 1000 | 0.96 |  |
| VOC9 | Tetradecane / nonadecane | 0.30809 | 0.07124 | 0.57179 | 1000 | 0.022 | * |
| VOC10 | Thiophene, 2,4-bis(1,1-dimethylethyl)- | -0.17719 | 0.85895 | 0.48167 | 1000 | 0.642 |  |
| VOC11 | 3-Tetradecyne /Hexadecyne | -0.3287 | 0.9506 | 0.4716 | 1000 | 0.376 |  |
| VOC12 | b - Chamigrene | -0.2008 | 0.9278 | 0.4635 | 1000 | 0.58 |  |
| VOC13 | E-1,9-Tetradecadiene | -2.439 | 6.808 | 1.657 | 1000 | 0.28 |  |
| VOC14 | Thujopsene | -0.06656 | 1.29774 | 1.00687 | 1000 | 0.93 |  |
| VOC15 | Tetradecane | 0.29613 | 0.26042 | 0.84615 | 1220 | 0.288 |  |
| VOC16 | b -Vatirenene | 6.282 | 2.698 | 14.571 | 1000 | 0.15 |  |
| VOC17 | Junipen | -3.227 | 13.46 | 5.618 | 1126.9 | 0.514 |  |
| VOC18 | b - Caryophyllene | 11.9331 | 0.5024 | 23.3222 | 1000 | 0.046 | * |
| VOC19 | Aromadendrene | -0.23101 | 0.98882 | 0.51291 | 1000 | 0.548 |  |
| VOC20 | Isosativene | 1.00634 | 0.02282 | 2.05212 | 1000 | 0.06 | . |
| VOC21 | a- Caryophyllene | 0.4555 | 13.1702 | 17.3942 | 903.2 | 0.94 |  |
| VOC22 | t-Muurolene | 1.3765 | 5.3947 | 2.3336 | 1000 | 0.486 |  |
| VOC23 | t-Muurolene | 0.2969 | 0.24546 | 0.79991 | 1000 | 0.258 |  |
| VOC24 | t- Gurjunene | -0.3148 | 1.1997 | 0.443 | 1000 | 0.456 |  |
| VOC25 | d - Cadinene | 0.32495 | 0.228217 | 0.944618 | 1000 | 0.316 |  |
| VOC26 | Nonadecane | 0.30365 | 0.88082 | 0.34051 | 1108 | 0.336 |  |
| Tot VOCs |  | 137.27 | 43.49 | 233.79 | 955.7 | 0.006 | ** |
| H |  | 0.09965 | 0.21231 | 0.01627 | 1093.1 | 0.088 | ° |

Signif. codes: 0 '***' 0.001 '**' 0.01 '*' 0.05 '.' 0.1 '°' 1

**Table S5**. Phylogenetic signal table. Shown are kappa (k) values and simulated p-values (p) for number of nematodes recruited, and all individual VOCs, total amount of VOCs (sum), the diversity of VOCs (H), as well as the eight climatic variables characterizing the niche of the 18 species of *Festuca.* Bold indicates significant phylogenetic signal.

|  | Control | | | Induced | | |  | Climate | | |
| --- | --- | --- | --- | --- | --- | --- | --- | --- | --- | --- |
|  | k | p |  | k |  |  |  | k | P |  |
| Nematode | 0.26 | 0.36 |  | 0.26 | 0.37 |  | **Radiation** | **0.66** | **0.01** | ** |
| VOC1 | 0.21 | 0.64 |  | 0.21 | 0.65 |  | **Elevation** | **0.45** | **0.05** | ** |
| VOC2 | 0.12 | 0.82 |  | 0.12 | 0.8 |  | Degree-days | 0.39 | 0.1 |  |
| VOC3 | 0.26 | 0.52 |  | 0.26 | 0.49 |  | **Evapotranspiration** | **0.49** | **0.03** | ** |
| VOC4 | 0.32 | 0.37 |  | 0.32 | 0.35 |  | Precipation-days | 0.35 | 0.15 |  |
| VOC5 | 0.29 | 0.38 |  | 0.29 | 0.36 |  | Frost-days | 0.38 | 0.14 |  |
| VOC6 | 0.32 | 0.27 |  | 0.32 | 0.24 |  | **Climatic niche (PCA1)** | **0.42** | **0.06** | ° |
| VOC7 | 0.23 | 0.56 |  | 0.23 | 0.56 |  | **Elevational range** | **0.46** | **0.04** | ** |
| VOC8 | 0.3 | 0.31 |  | 0.3 | 0.32 |  |  |  |  |  |
| **VOC9** | **1.04** | **0.08** | ° | **1.04** | **0.09** | ° |  |  |  |  |
| **VOC10** | **0.43** | **0.09** | ° | **0.43** | **0.09** | ° |  |  |  |  |
| VOC11 | 0.34 | 0.35 |  | 0.34 | 0.32 |  |  |  |  |  |
| VOC12 | 0.17 | 0.79 |  | 0.17 | 0.75 |  |  |  |  |  |
| VOC13 | 0.19 | 0.7 |  | 0.19 | 0.69 |  |  |  |  |  |
| VOC14 | 0.33 | 0.22 |  | 0.33 | 0.23 |  |  |  |  |  |
| VOC15 | 0.18 | 0.74 |  | 0.18 | 0.74 |  |  |  |  |  |
| VOC16 | 0.22 | 0.58 |  | 0.22 | 0.61 |  |  |  |  |  |
| VOC17 | 0.26 | 0.49 |  | 0.26 | 0.5 |  |  |  |  |  |
| VOC18 | 0.16 | 0.74 |  | 0.16 | 0.76 |  |  |  |  |  |
| VOC19 | 0.07 | 0.97 |  | 0.07 | 0.96 |  |  |  |  |  |
| VOC20 | 0.15 | 0.79 |  | 0.15 | 0.79 |  |  |  |  |  |
| VOC21 | 0.13 | 0.81 |  | 0.13 | 0.79 |  |  |  |  |  |
| VOC22 | 0.19 | 0.74 |  | 0.19 | 0.71 |  |  |  |  |  |
| VOC23 | 0.23 | 0.54 |  | 0.23 | 0.56 |  |  |  |  |  |
| VOC24 | 0.22 | 0.64 |  | 0.22 | 0.62 |  |  |  |  |  |
| VOC25 | 0.22 | 0.58 |  | 0.22 | 0.6 |  |  |  |  |  |
| VOC26 | 0.27 | 0.39 |  | 0.27 | 0.37 |  |  |  |  |  |
| **sum** | **0.45** | **0.07** | ° | **0.60** | **0.05** | ** |  |  |  |  |
| H_vocs | 0.31 | 0.23 |  | 0.31 | 0.21 |  |  |  |  |  |

Significance codes: ° = < 0.01, ** = < 0.05.

Significant p-values indicate phylogenetic signal (k is different from 0).

**Supplementary Figures**

**Figure S1.** General phylogeny, with species chosen for the bioassays and analyses highlighted with black arrows.


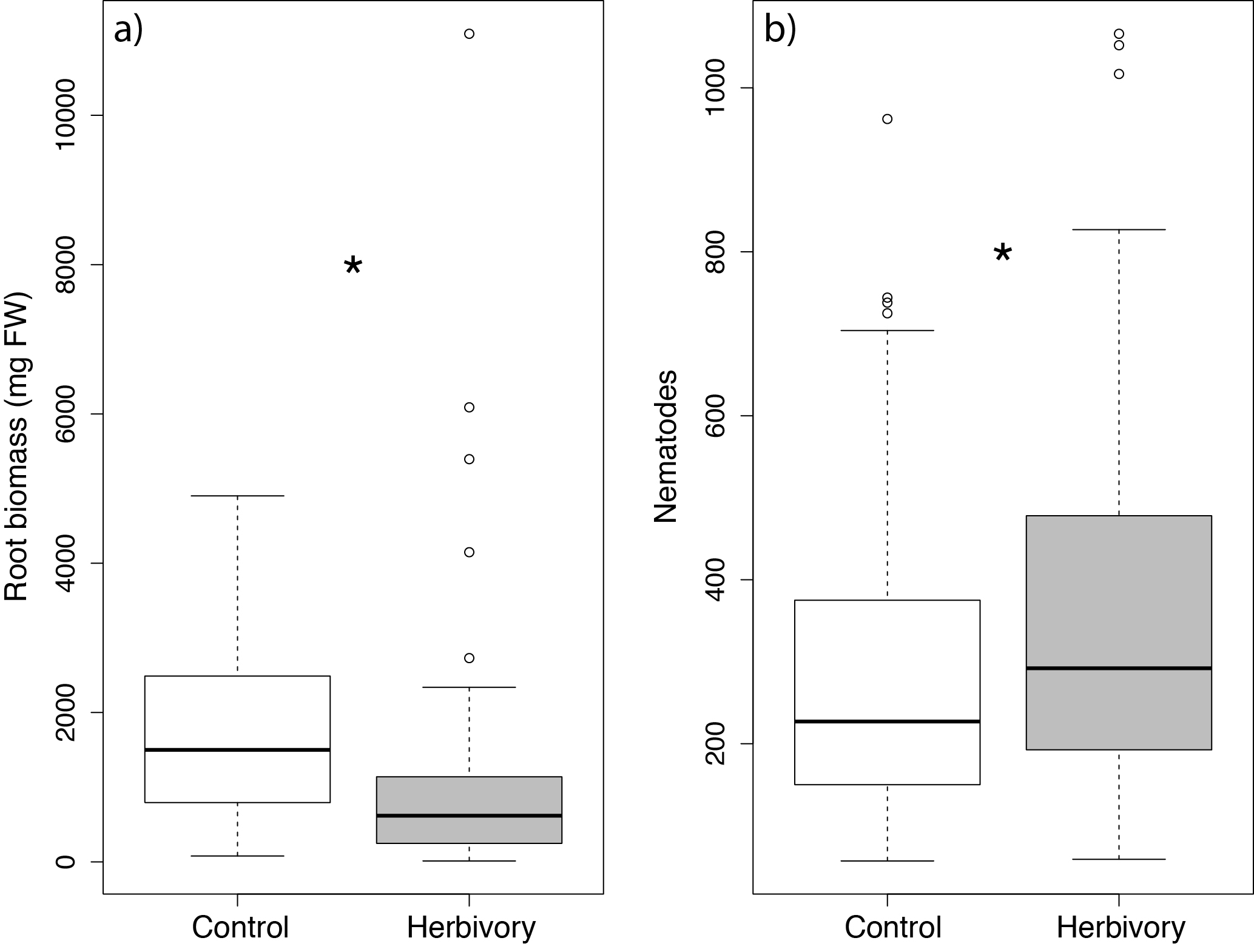


**Figure S2**. Root biomass loss and nematodes attracted as a function of herbivore treatment averaged across 18 *Festuca* species (MCMCglmm for root biomass; post.mean = -657.9, l-95% CI = -1090.2, u-95% CI = -265.9, sampling = 1000, pMCMC = 0.002. MCMCglmm for nematodes; post.mean = 96.49, l-95% CI = 31.81, u-95% CI =160.60, effect sampling = 868, pMCMC = 0.002). Asterisks above boxes indicate significant difference among herbivore treatments (p < 0.05).


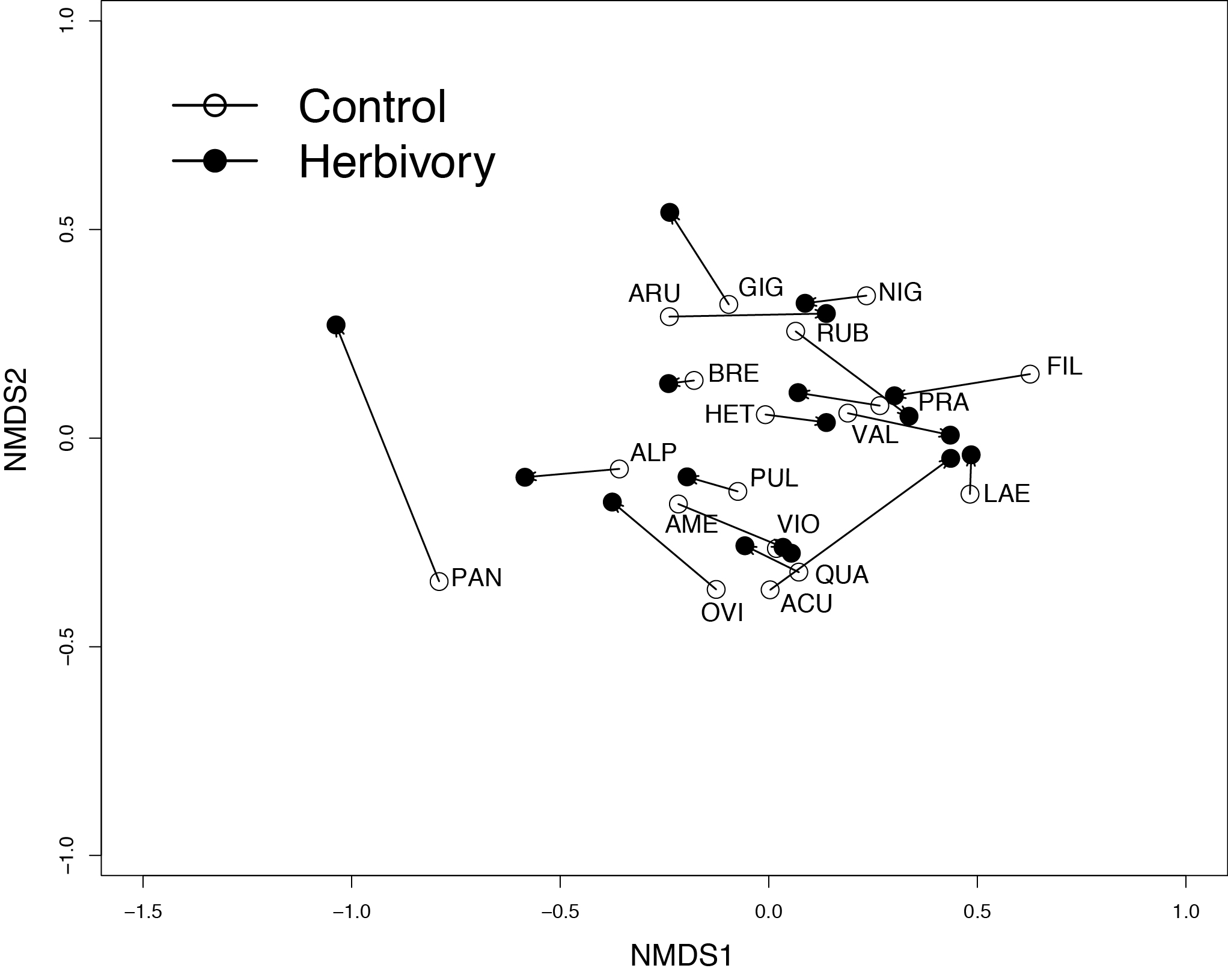


**Figure S3**. nMDS plot of VOCs produced by 18 species of *Festuca* in the absence (Control, open dots), or after *Melolontha melolontha* root herbivore attack (Herbivory, black dots). Lines rely the same species that experienced both the control and herbivore treatments. ACU = *F. acuminata,* ALP = *F. alpina*; AME = *F. amethystina*; ARU = *F. arundinacea*; BRE = *F. brevipila*; FIL = *F. filiformis*; GIG = *F. gigantea*; HET = *F. heterophylla*; LAE = *F. laevigata*; NIG = *F. nigrescens*; OVI = *F. ovina*; PAN = *F. paniculata*; PRA = *F. pratensis*; PUL = *F. pulchella*; QUA = *F. quadriflora*; RUB = *F. rubra*; VAL = *F. valesiaca*; VIO = *F. violacea*. nMDS stress = 0.2.


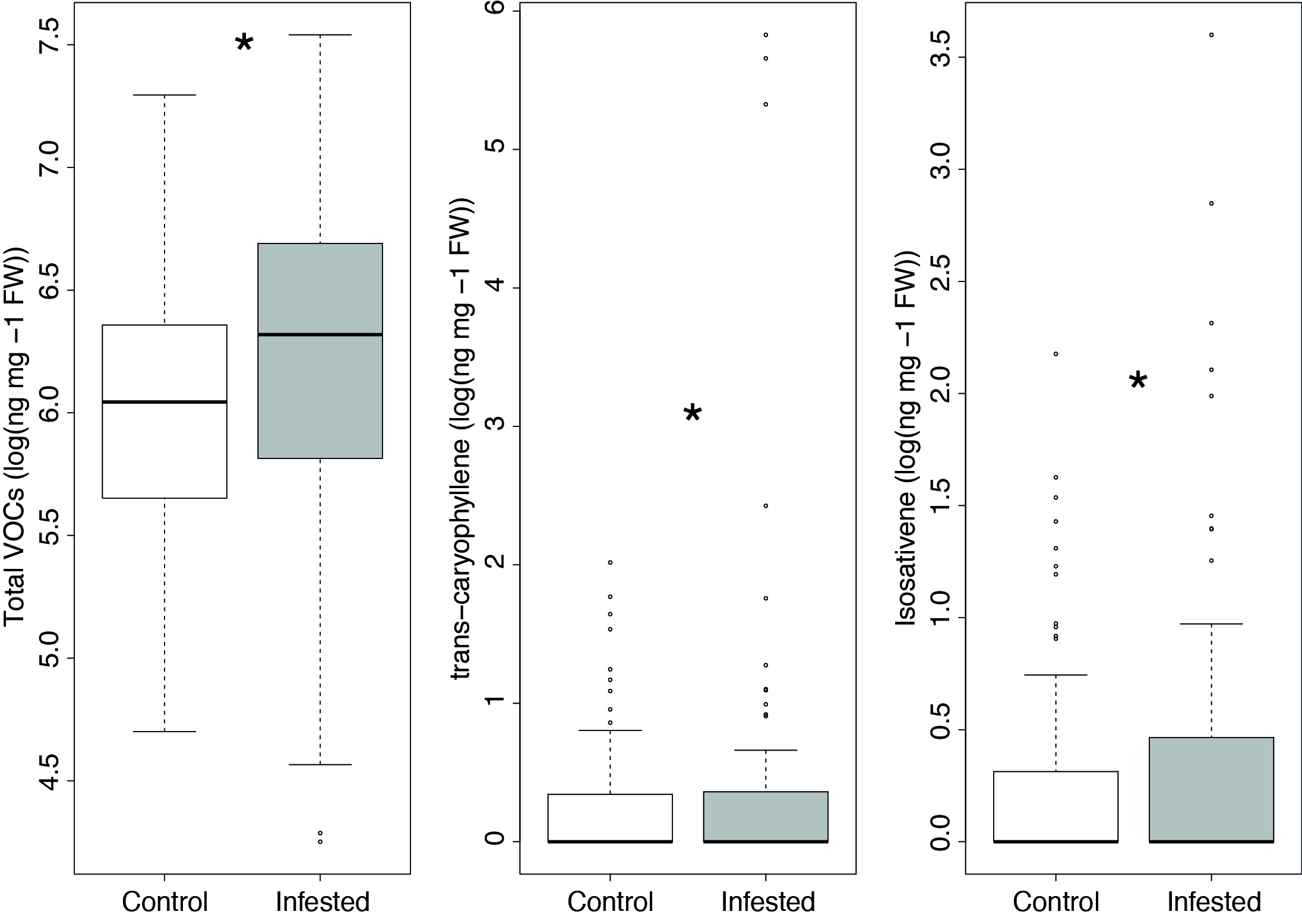


**Figure S4.** Induction of VOCs after root herbivory. Shown are from left to right, total amount of VOCs produced, trans-caryophyllene and isosativene, two sesquiterpendoids VOCs. Asterisks above boxes indicate significant difference among herbivore treatments ((MCMCglmm ; p < 0.05).

**Figure S5.** Correlation between constitutive root volatile organic compound (VOCs C) emission and their inducibility (VOCs I-C). Dots are color-coded based on each species optimal elevation niche (see Fig. S11); warmer (red) color represent warmer climates, and colder (blue) colors represent colder climates.

**Figure S6.** PCA of climate variables. For each of the 18 species of *Festuca*, we quantified the realized climatic niche in Switzerland using the occurrence datapoints obtained from the National Data Center and Information on the Flora of Switzerland (www.infoflora.ch). Occurrence data varied between a minimum of 25 points for rare species such as *F. brevipila* to more than 2000 points for common species such as *F. rubra* or *F. ovina.* Values for degree-days and potential evapotranspiration, precipitation, and frost days indices were calculated from meteorological stations combined with a Digital Elevation Model (DEM) at 100 m resolution, and interpolated following ^8^. Solar radiation values were estimated using the tool implemented in ArcGIS 10. ddeeg300 = degree-days; pday = number of precipitation days per growing season; mind59 = potential evapotranspiration; mnt25 = elevation (m); sfroyy = yearly average number of frost days during growing season; srad = average solar radiation.


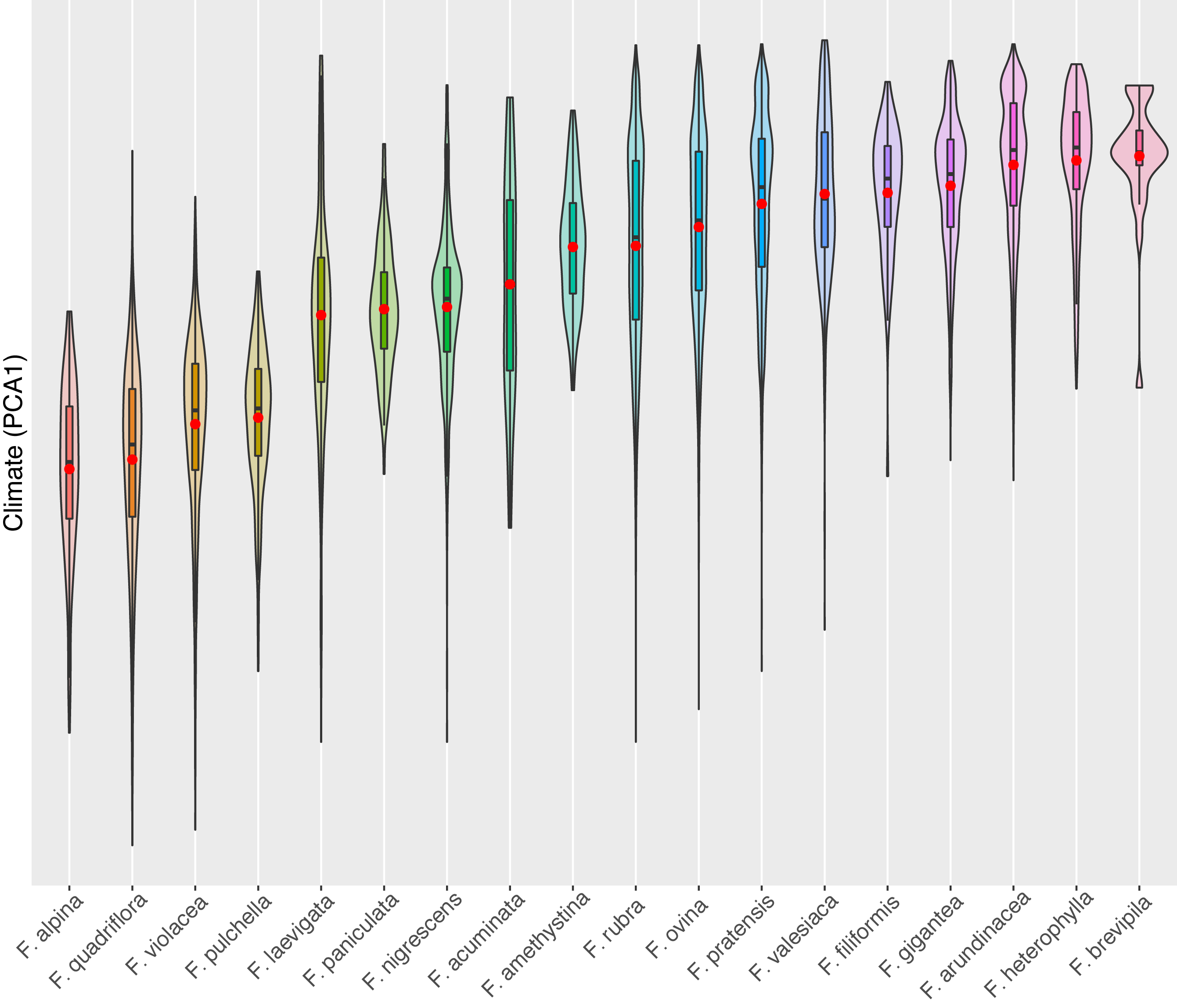


**Figure S7.** Organization of each of the 18 sampled *Festuca* species along the first axis of the climatic PCA (as in Figure S3). The PCA axis goes from cold and humid to warm and dry. Violin plots represent the magnitude of observations of each species at each climatic range.


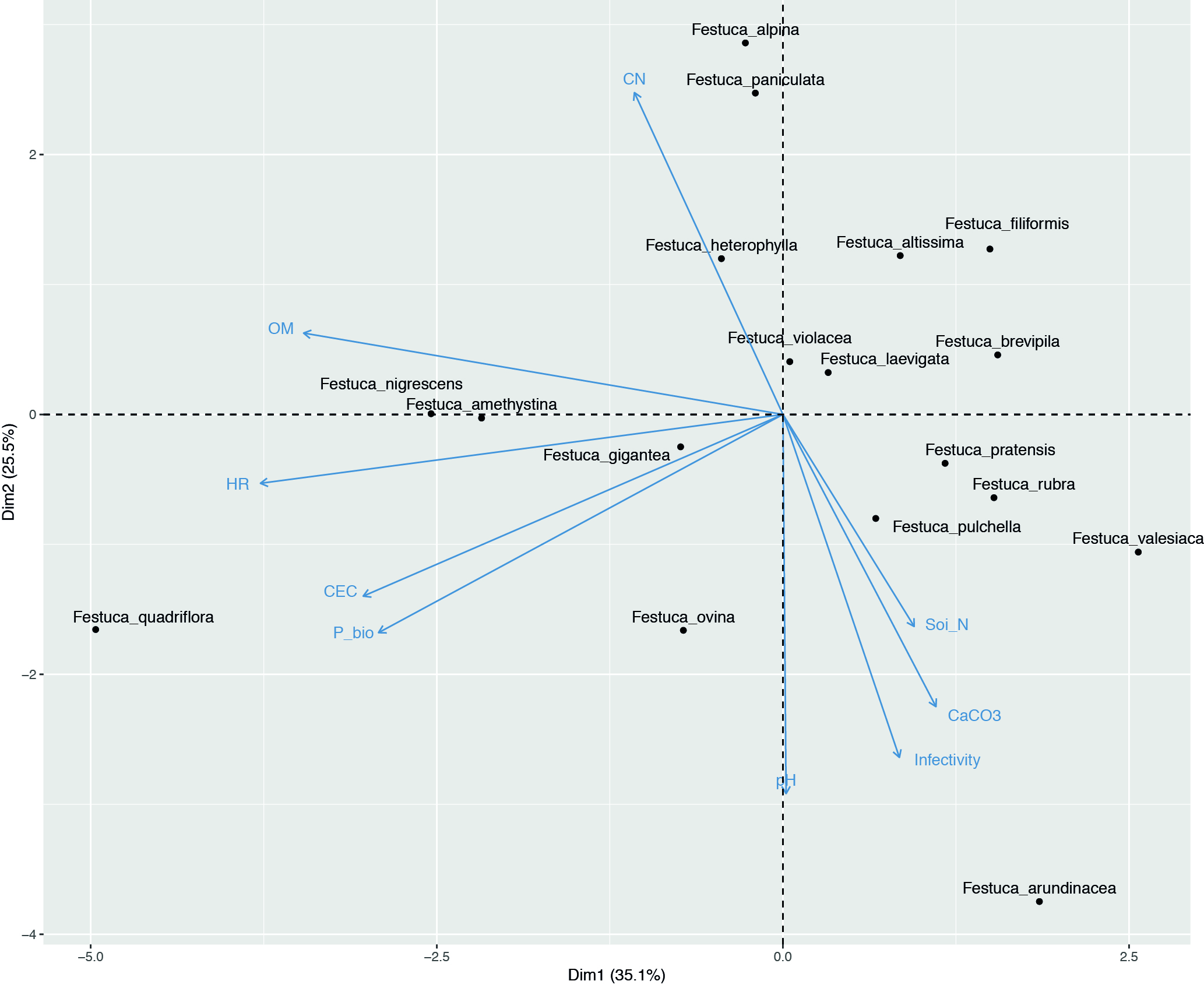


**Figure S8.** PCA of soil variables. Soil variables are: Soil_N = Landolt value of soil fertility; CaCO3 = total carbonates; Infectivity = EPN infectivity potential on *Galleria mellonella* waxworms; pH = pH in water; P_bio = bioavailable phosphorous; CEC = cationic exchange capacity; HR = soil relative humidity; OM = total soil organic matter after combustion; CN = organic carbon to total nitrogen ratio. See average values per species in Table S3.

**Figure S9**. Distance-based redundancy analysis (dbRDA) for measuring the correlation between the herbivore-induced VOCs matrix and the soil variables matrix. The model show significant association between the two matrices (F_8,9_ = 1.67, p = 0.01; CCA1: Chi2 = 0.59, p = 0.04; CCA2: Chi2 = 0.38, p = 0.09). Of all variable, HR: Chi2 = 0.32, p = 0.02; pH: Chi2 = 0.31, p = 0.01; CEC: Chi2 = 0.33, p = 0.01; and CN: Chi2 = 0.26, p = 0.02 were best explaining the VOCs matrix.


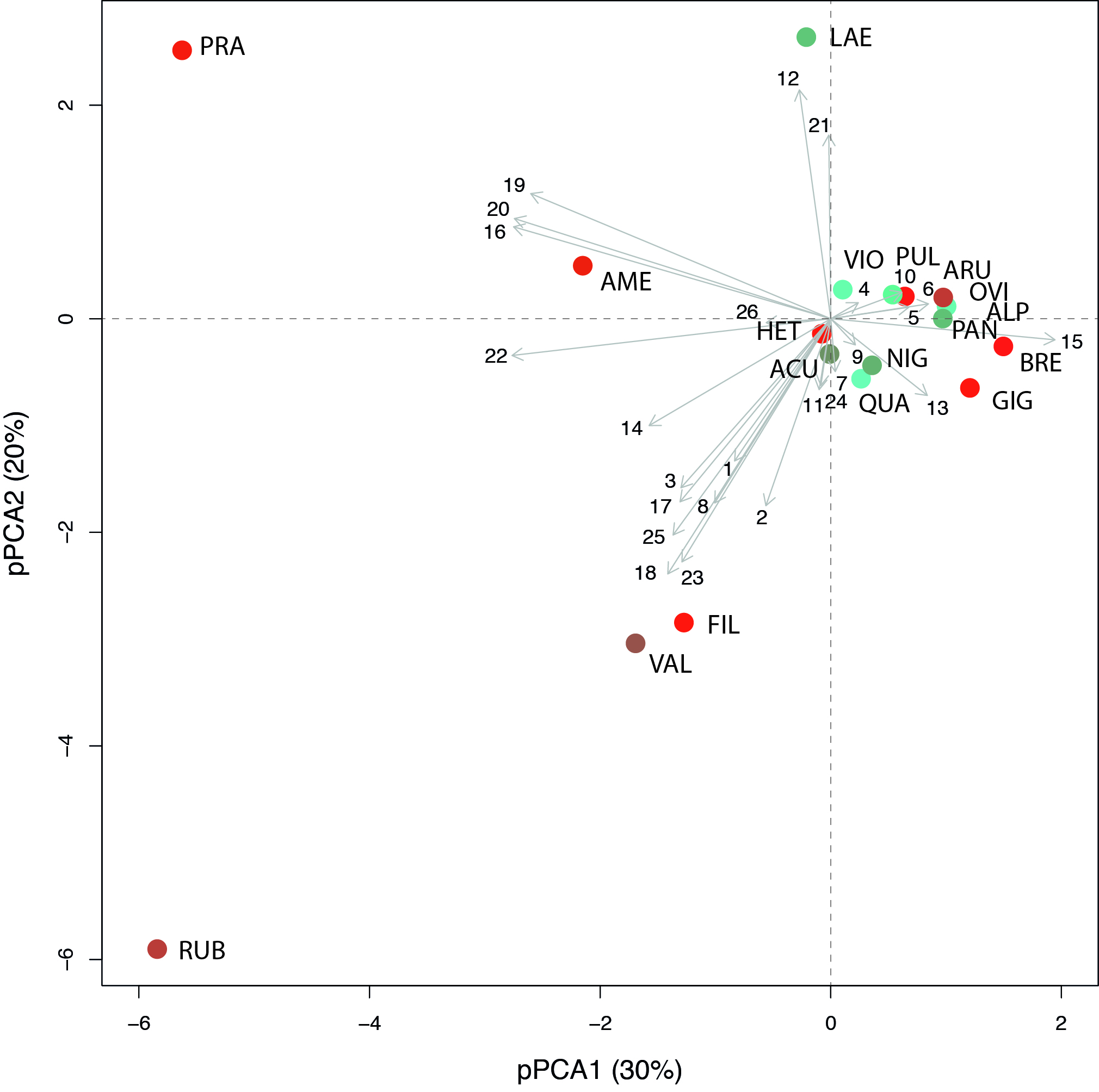


**Figure S10.** Phylogenetic PCA (pPCA) of induced VOCs across 18 species of *Festuca*. ACU = *F. acuminata,* ALP = *F. alpina*; AME = *F. amethystina*; ARU = *F. arundinacea*; BRE = *F. brevipila*; FIL = *F. filiformis*; GIG = *F. gigantea*; HET = *F. heterophylla*; LAE = *F. laevigata*; NIG = *F. nigrescens*; OVI = *F. ovina*; PAN = *F. paniculata*; PRA = *F. pratensis*; PUL = *F. pulchella*; QUA = *F. quadriflora*; RUB = *F. rubra*; VAL = *F. valesiaca*; VIO = *F. violacea.*


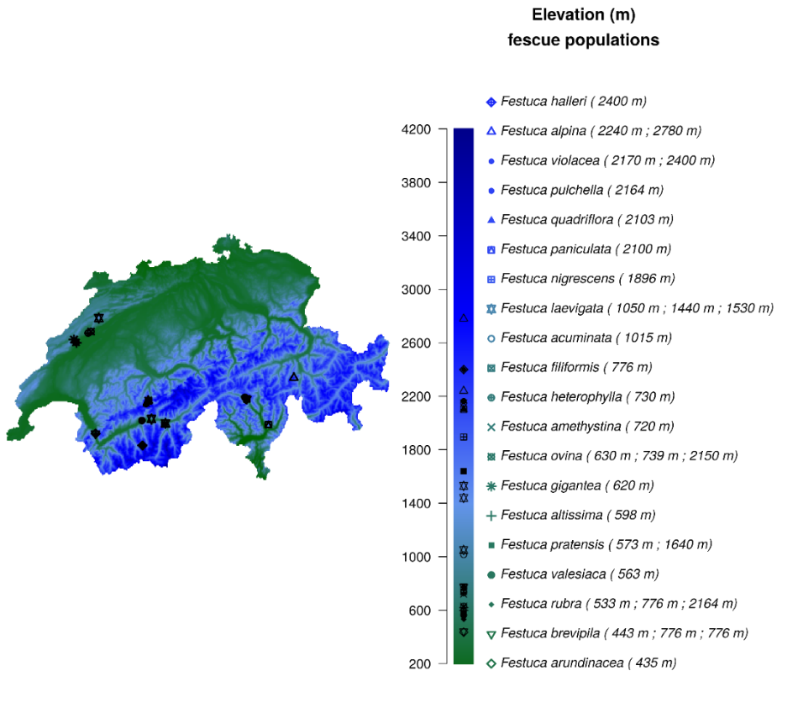


**Figure S11.** Locations of *Festuca* plant species collection sites and average distribution range of each species.

**Figure S12.** Cladogram of the Best Maximum Likelihood nuclear ITS tree of *Festuca* and outgroup samples computed with IQTREE. *Brachypodium distachyon* was used to root the tree. Boostrap support (BS) values are indicated on branches. Main phylogenetic lineages of the studied Loliinae (*Festuca*) are indicated on its right. The positions of *F. acuminata*, *F. nigrescens*, *F. brevipila* and *F. filiformis* are highlighted in blue. The ITS ML IQTREE and Bayesian BEAST trees recovered overall highly congruent topologies that had well supported branches for the main *Festuca* clades. The Loliinae clade was relatively well supported in both trees [88% bootstrap support (BS); 0.94 posterior probability support (PPT)], showing the successive divergences of the broad-leaved Drymanthele and Leucopoa and the Subbulbosae and Schedonorus lineages, followed by those of the fine-leaved Eskia-Dimorpha, Exaratae and Aulaxyper and Festuca lineages. In both trees *F. capillifolia* was resolved as sister of the Festuca clade. The newly sequenced *F. acuminata, F. nigrescens*, and *F. brevipila* and *F. filiformis* fell, respectively, within the Eskia-Dimorpha (0.84 PPS), the Aulaxyper (100BS, 1 PPS) and the Festuca (100BS, 1 PPT) clades. *F. acuminata* was resolved as sister of *F. pumila* (87 BS), *F. nigrescens* as member of the core *F. rubra* group (96 BS, 0.73 PPT), and *F. brevipila* and *F. filiformis* as members of the *F. laevigata*-*F. panciciana* group (97 BS, 1 PPT). IQTREE analyses were done by imposing the best-fit nucleotide substitution model of each data set (ITS: TIM2e+G4; trnTLF: K3Pu+F+R2) that were selected by the ModelFinder option of the program according to the Bayesian Information Criterion (BIC). The respective automated searches consisted in the computation of 20 ML starting trees from 98 alternative randomized Maximum Parsimony (MP) trees, searching for best-scoring ML trees and estimating branch support for the best tree from 1,000 bootstrap replicates using the ultrafast bootstrap option implemented in the software.

**Figure S13**. Bayesian Maximum Clade Credibility (MCC) nuclear ITS cladogram of *Festuca* and outgroup samples computed with BEAST. *Brachypodium distachyon* was used to root the tree. Estimated divergence times (Ma) are indicated on branches; nodal bars represent 95% High Posterior Density (HPD) intervals. Bottom scale represent Ma. The positions of *F. acuminata*, *F. nigrescens*, *F. brevipila* and *F. filiformis* are highlighted in blue. Bayesian phylogenetic analysis were performed in BEAST v. 1.8.0 ^9^ imposing independent site substitution models close to those selected before for each data set (ITS: GTR+gamma; trnTFL: HKY), lognormal relaxed clock and Yule tree models, a broad uniform distribution prior for the uncorrelated lognormal distribution (ucld) mean (lower = 1.0E-6; upper = 0.1) and an exponential prior for ucld standard deviation (SD) to each data set. In order to infer the ancestral divergence ages of the *Festuca* lineages we used two calibration points, imposing normal distribution secondary age constrains for the crown nodes of the *Brachypodium* + core pooids clade (normal prior mean = 31.18 Ma, SD =3.5) and the core pooids clade [*Secale* + *Poa/Festuca*] (normal prior mean = 29.68 Ma, SD =3.5) following the grass-wide plastome based dating analysis of Sancho et al. ^10^. One hundred Markov chain Monte Carlo (MCMC) generations were run in BEAST with a sampling frequency of 1000 generations. The adequacy of parameters was checked using TRACER v.1.6 (http://beast.bio.ed.ac.uk/Tracer) with all parameters showing Effective Sample Size (ESS) >200. Maximum clade credibility (MCC) trees were computed after discarding 10% of the respective saved trees as burn-in. Divergence ages of the main *Festuca* lineages in the dated ITS BEAST Maximum Clade Credibility (MCC) tree were in general agreement with those retrieved in the large studies of Loliinae by Inda et al. ^1^ and Minaya et al. ^2^. The split of the crown node of Loliinae was estimated to have occurred in the early-mid Miocene (16.7 Ma), and those of other *Festuca* clades in the mid-late Miocene (fine-leaved, 10.0 Ma), the late Miocene (Eskia-Dimorpha core clade, 5.3 Ma), and the Pliocene (*F. rubra*, 4.0 Ma, and *F. ovina*, 3.8 Ma, core groups). The divergences of younger intra-clade lineages of the Eskia, *F. rubra* and *F. ovina* groups spanned the Pliocene and the Quaternary.

**Figure S14**. Cladogram of Best Maximum Likelihood plastid trnTL+trnLF tree of *Festuca* and outgroup samples computed with IQTREE. *Brachypodium distachyon* was used to root the tree. Boostrap support (BS) values are indicated on branches. The positions of *F. acuminata*, *F. brevipila* and *F. filiformis* are highlighted in blue, and that of *F. nigrescens* in red. The trnTL+trnLF ML IQTREE and BEAST Bayesian trees recovered topologies highly congruent to each other, though they differed in the positions of several sequences with respect to the ITS topologies. In the two plastid trees the Loliinae clade was highly supported (100 BS, 1 PPT) and showed a monophyletic and well supported broad-leaved clade (98 BS, 1 PPT), the intermediate position of *F. pulchella*, a paraphyletic Eskia-Dimorpha group, and the recent divergences of the strongly supported Exaratae (including *F. capillifolia* and *F. amethystina*) (100 BS, 1 PPT) and the Aulaxyper (100 BS, 1 PPT) and Festuca (100 BS, 1 PPT) clades. *Festuca acuminata* was resolved as sister to *F. pumila* (83 BS) and *F. brevipila* and *F. filiformis* as members of a large *F. ovina* group (96 BS, 1 PPT). Noticeably, *F. nigrescens* was nested within the well-supported *F. ovina* group.

**Literature cited:**

1 Inda, L. A., Segarra-Moragues, J. G., Muller, J., Peterson, P. M. & Catalan, P. Dated historical biogeography of the temperate Loliinae (Poaceae, Pooideae) grasses in the northern and southern hemispheres. *Mol. Phylogen. Evol.* **46**, 932-957, doi:10.1016/j.ympev.2007.11.022 (2008).

2 Minaya, M. *et al.* Contrasting dispersal histories of broad- and fine-leaved temperate Loliinae grasses: range expansion, founder events, and the roles of distance and barriers. *J. Biogeogr.* **44**, 1980-1993, doi:doi:10.1111/jbi.13012 (2017).

3 Hand, M. L., Spangenberg, G. C., Forster, J. W. & Cogan, N. O. I. Plastome sequence determination and comparative analysis for members of the *Lolium-Festuca* grass species complex. *G3 (Bethesda, Md.)* **3**, 607-616, doi:10.1534/g3.112.005264 (2013).

4 Paradis, E., Claude, J. & Strimmer, K. APE: analyses of phylogenetics and evolution in R language. *Bioinformatics* **20**, 289-290, doi:10.1093/bioinformatics/btg412 (2004).

5 Markgraf-Dannenberg, I. in *Flora Europaea* Vol. 5 (eds T. G. Tutin *et al.*) 125-153 (Cambridge University Press, 1980).

6 Auquier, P. & Rammeloo, J. Nombres chromosomiques dans le genre *Festuca* en Belgique et dans les régions limitrophes. *Bulletin de la Société Royale de Botanique de Belgique / Bulletin van de Koninklijke Belgische Botanische Vereniging* **106**, 317-328 (1973).

7 Aeschimann, D., Lauber, K., Moser, D. M. & Theurillat, J.-P. *Flora Alpina*. (Haupt 2004).

8 Zimmermann, N. & Kienast, F. Predictive mapping of alpine grasslands in Switzerland: species versus community approach. *Journal of Vegetation Science* **10**, 469-482 (1999).

9 Drummond, A. J., Suchard, M. A., Xie, D. & Rambaut, A. Bayesian phylogenetics with BEAUti and the BEAST 1.7. *Mol. Biol. Evol.* **29**, 1969-1973, doi:10.1093/molbev/mss075 (2012).

10 Sancho, R. *et al.* Comparative plastome genomics and phylogenomics of *Brachypodium:* flowering time signatures, introgression and recombination in recently diverged ecotypes. *New Phytol.* **218**, 1631-1644, doi:10.1111/nph.14926 (2018).
